# Innovative Bioactive Products with Medicinal Value from Microalgae and Their Overall Process Optimization through the Implementation of Life Cycle Analysis—An Overview

**DOI:** 10.3390/md22040152

**Published:** 2024-03-28

**Authors:** Sofia Papadaki, Nikoletta Tricha, Margarita Panagiotopoulou, Magdalini Krokida

**Affiliations:** 1 DIGNITY Private Company, 30-32 Leoforos Alexandrou Papagou, Zografou, 157 71 Athens, Greece; 2 Laboratory of Process Analysis and Design, School of Chemical Engineering, National Technical University of Athens, Iroon Polytechneiou 9, 157 80 Athens, Greece; nikoletta.tricha@gmail.com (N.T.); panagiot.marg@gmail.com (M.P.); mkrok@chemeng.ntua.gr (M.K.)

**Keywords:** microalgae, carotenoids, phycobiliproteins, bioactive peptides, ω-3 fatty acids, novel extraction techniques, encapsulation, sustainability assessment

## Abstract

Microalgae are being recognized as valuable sources of bioactive chemicals with important medical properties, attracting interest from multiple industries, such as food, feed, cosmetics, and medicines. This review study explores the extensive research on identifying important bioactive chemicals from microalgae, and choosing the best strains for nutraceutical manufacturing. It explores the most recent developments in recovery and formulation strategies for creating stable, high-purity, and quality end products for various industrial uses. This paper stresses the significance of using Life Cycle Analysis (LCA) as a strategic tool with which to improve the entire process. By incorporating LCA into decision-making processes, researchers and industry stakeholders can assess the environmental impact, cost-effectiveness, and sustainability of raw materials of several approaches. This comprehensive strategy will allow for the choosing of the most effective techniques, which in turn will promote sustainable practices for developing microalgae-based products. This review offers a detailed analysis of the bioactive compounds, strain selection methods, advanced processing techniques, and the incorporation of LCA. It will serve as a valuable resource for researchers and industry experts interested in utilizing microalgae for producing bioactive products with medicinal properties.

## 1. Introduction

Compounds of natural origin have been used for thousands of years as a means of disease prevention and therapy, and seen in many of the ancient, developed civilizations. Sumerians used thyme medicinally from 5000 BC, while Indian Ayurveda focused on nutrition, with basil, cinnamon, and ginger used for their various health benefits. Later, Greek physicians like Hippocrates and Galen utilized garlic, rosemary, and complex remedies for treating ailments [1,2]. The idea of the so-called “nutraceuticals” has always been prominent, with strong evidence from scientific research highlighting the need for bioactive compounds to be incorporated into the human diet, as well as pharmaceutics, such as carotenoids, amino acids, polyphenols, and polyunsaturated fatty acids from plants, fungi, microbes, or animals that possess strong antioxidant, anticancer, and health protective effects for different organ systems [3,4,5,6]. As of 2022, the bioactive compounds market has exhibited its potential, reaching USD 173.3 billion, with a projected lucrative growth of USD 302.3 billion by 2030 [7]. However, within the scope of modern demands for medicine and nutrition convergence, the exploitation of natural sources should proceed sustainably and ethically, with the ultimate goal being the minimum strain on the ecosystem by land and resource preservation, as well as the maximum benefit accumulation. This way, the constantly increasing population’s needs could be more sufficiently fulfilled in an inclusive and indivisible manner. To achieve this multifactorial scenario, innovation and research should focus on the pursuit of alternative nutraceutical resources and the optimization of the processes used to yield them.

As far as alternative resources are concerned, microalgae are considered a key player in nutraceutical production. Algae have existed on Earth for approximately 3.5 billion years, surviving in diverse climate conditions, mainly in coastal and aquatic ecosystems [8]. Microalgae are a highly diversified species with more than 30,000 identified strains. Due to their unique properties, especially their ability to capture CO_2_ during cultivation, they could serve as an excellent alternative raw material with a low carbon footprint. They also have a short life cycle, which is characterized by a great growth rate when compared to terrestrial plants, resulting in a higher biomass accumulation, while requiring minimal land and water use, contributing to the sustainability of their utilization [8,9]. By manipulating their external stressors and cultivation conditions and media, microalgae could produce a variety of bioactive metabolites, including carotenoids, vitamins, phycobiliproteins, and ω-3 fatty acids, which could later be incorporated into functional foods [10].

Central to the commercialization of microalgae-derived bioactive compounds is the development of efficient processes that preserve their biological activities and structural integrity. The extraction process is critical for liberating the target molecules from the microalgae biomass, while it presents inherent challenges due to the rigid cell wall structure and complex intracellular organization of microalgae cells. Conventional extraction techniques, although successful, usually involve large energy inputs, pose environmental issues since they use large quantities of solvents possibly harmful for humans and the environment, and may cause thermally unstable substances to degrade, thus limiting the process scale-up [11]. Novel extraction technologies, such as ultrasound-assisted extraction, m-assisted extraction, and pressurized liquid extraction, have gained popularity in academic research due to their improved selectivity, efficiency, and sustainability [12]. Following extraction, the bioactive compounds require encapsulation to enhance their stability, bioavailability, and targeted delivery. Encapsulation technologies play a pivotal role in protecting the bioactives from degradation, improving their solubility, and facilitating their controlled release kinetics [13]. Electrospinning and spray–freeze drying are considered state-of-the-art encapsulation processes of great interest for nutraceutical production, due to their versatility and wide operation applicability [14].

Life Cycle Analysis (LCA) plays a pivotal role in optimizing microalgae-based processes for sustainability and efficiency. LCA provides a comprehensive framework for assessing the environmental impacts associated with the entire life cycle of microalgae-derived bioactive products, from their cultivation and extraction to product utilization and disposal. By quantifying resource consumption, energy usage, emissions, and other environmental indicators at each stage of the production process, LCA enables stakeholders to identify process hotspots, compare techniques, evaluate trade-offs, and implement targeted improvements, aiding in the selection of methods that minimize the environmental impact while maximizing product quality and yield. Ultimately, the integration of LCA into microalgae-based processes will promote sustainability by guiding the development of eco-friendly practices and fostering the transition towards a circular bioeconomy [15,16].

This study focuses on the presentation of a literature review of the most valuable bioactive compounds from microalgae, as well as the identification of the most suitable strains for nutraceutical production. Thereafter, this review will explore the most innovative recovery and final formulation techniques for stable, high-purity, and quality end products that will be used as ingredients in the food, feed, cosmetics, and pharmaceutical industries. Finally, the last part of this study focuses on the importance of Life Cycle Analysis as a decision-making tool that will support researchers and industries in selecting the most efficient techniques in terms of environmental impact, cost methodology, and raw materials.

## 2. Bioactive Compounds of Marine Origin

Microalgae have emerged as a promising source of bioactive compounds, attracting significant interest from both industry and academia. Microalgae are unicellular organisms that are widely found in different aquatic ecosystems. They have a rich content of bioactive compounds (fatty acids, carotenoids, phenols, vitamins, phycobiliproteins, and pigments) that have various health benefits and can be used in pharmaceuticals, functional foods, feed, and nutraceuticals. It is important to note that although the discovery of the bioactive compound variety within microalgae offers a promising avenue for medicinal and nutritional research, studies on microalgae have usually not prioritized compounds such as polyphenols and vitamins, which are frequently plentiful in terrestrial plants. Microalgae often have lower amounts of polyphenolic compounds than their plant counterparts [17]. Rather, the focus has centered around substances that are particularly plentiful, bioactive and even, occasionally, unique in microalgal species, such as phycobiliproteins and their pigments, carotenoids, and ω-3 fatty acids [18,19,20,21]. This strategic focus guarantees that efforts by industry and researchers are directed toward maximizing the potential of the bioactive compounds obtained from microalgae that have the greatest effects on human health and sustainable nutrition, serving as a great process scale-up opportunity with commercial interest.

### 2.1. Carotenoids

Carotenoids are a class of pigmented terpenoid compounds with a backbone skeleton of 40 carbon atoms, consisting of eight isoprene molecules, from which every variation is derived. Currently, more than 600 compounds have been identified and characterized with unique functional properties. Typically recognized as plant pigments, manifested by the colors of yellow, orange, and red fruits and flowers, they also supply animals and microbes with their vivid hues. They constitute crucial elements of oxygenic photosynthesis because of their other, less evident functions. Photosynthesis and all forms of life in an aerobic environment would be impossible without carotenoids [22,23].

Carotenoid biosynthesis occurs in the chloroplasts of higher plants, bacteria, fungi, and algae, while they become accessible to animals through nutrition [24]. As the key components of light-harvesting complexes in photosynthetic organisms, carotenoids play a vital role in the energy-harvesting process during photosynthesis. Their biosynthesis is a complex process incorporating several enzyme systems. The following key phases may be used to divide the entire biosynthetic process: (1) the synthesis of isopentenyl diphosphate (IPP) from glucose; (2) the enzymatic conversion of IPP to phytoene; (3) the desaturation (dehydrogenation) of phytoene to -carotene, neurosporene, and lycopene; (4) the cyclization and the formation of α-, and β-carotenes; (5) the hydroxylation and epoxidation of carotenes and the creation of the cyclic xanthophylls of chloroplasts [24,25].

**Figure 1:**
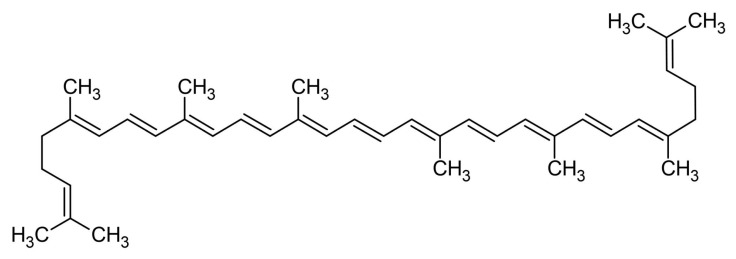
Lycopene is the main structure of carotenoids.

Figure 1 illustrates the main structure of carotenoids, called lycopene, an acyclic carotenoid. The middle region of carotenoid structures is characterized by a sequence of alternating single and double bonds, named the chromophore. The conjugated system’s π-electrons delocalize across the chromophore, causing light absorption in the visible spectrum and, therefore, the coloration of the tissues and carotenoids that contain them. Formally, all carotenoids may be produced by any combination of the following processes from the acyclic C_40_H_56_ structure: (i) hydrogenation, (ii) dehydrogenation, (iii) cyclization, or (iv) oxidation [22].

Every double bond in a carotenoid’s polyene chain has the potential to exist in either a trans or cis form, depending on how the substituent groups surrounding that double bond are arranged. This results in a vast number of theoretically feasible monocis and polycis isomers; yet, in practice, the majority of carotenoids are found mostly or exclusively in the linear all-trans form, due to the thermodynamic stability of the trans against the cis isomers in most cases [22,26]. The two main categorizations of carotenoids are the carotenes, consisting only of carbon and hydrogen atoms, i.e., lycopene, α-carotene, β-carotene and the xanthophylls, also containing oxygen atoms in the form of carbonyl, hydroxyl, keto, and epoxy groups (i.e., astaxanthin, lutein, and zeaxanthin) [24].

As they efficiently absorb excess energy, carotenoids inhibit the production of reactive oxygen species (ROS) and deactivate the singlet oxygen produced during photosynthetic processes, thus displaying a protective function in organisms. Additionally, carotenoids produce radical cations by electron transfer reactions with a variety of free radicals, including O_2_∙, RSO_2_∙, and NO_2_∙. Physical processes, in which the excess energy of singlet oxygen is transmitted to the carotenoid, have been primarily attributed to the quenching of singlet oxygen by carotenoids. Chemical interactions between excited oxygens and carotenoids are not as significant as physical quenching, accounting for less than 0.05 percent of the total quenching rate [22,26,27]. This trait against ROS has sparked a lot of research on the potential antioxidant properties of carotenoids and their consequent mechanisms, as well as the accumulation of the compounds from sustainable sources at a sufficient rate for nutritional and medicinal applications.

The worldwide carotenoid industry is expected to reach USD 2 billion by 2026, including food and beverages (26.1%), cosmetics (6.5%), dietary supplements (23.5%), and pharmaceuticals (9.2%) [28]. Most commercial carotenoid needs have, up till now, been met via chemical synthesis. However, due to growing concerns about safety and potential harmful repercussions, there is a greater need for natural sources of carotenoid synthesis. Due to their special qualities, microalgae have been discovered to be a viable feedstock for carotenoid accumulation, by adjusting natural variables like temperature, light, and pH during their cultivation period [29]. The most abundant carotenoids in microalgae are astaxanthin, mainly in *Heamatococcus pluvialis*, which is considered the main natural source of astaxanthin, β-carotene, lutein, and its isomer zeaxanthin in the *Spirulina* strains, and *Dunaliella salina* and *Chlorella* strains. The microalgae with significant contents of carotenoids are displayed in Table 1. 

Many of these strains have demonstrated health benefits because of their carotenoid contents. Rao et al. [33] examined the bioavailability and antioxidant properties of carotenoids from algal biomasses using a rat model. For fifteen days, the rats were administered a microalgal biomass that included 200 μM of lutein, astaxanthin, and β-carotene per rat, derived from the biomass of *Spirulina platensis*, *Haematococcus pluvialis*, and *Botryococcus braunii*, respectively. By high-performance liquid chromatography, the concentrations of those carotenoids were measured in the plasma, liver, and eye. The highest peak concentrations (nmol/g) of lutein (679.55 ± 74.08), astaxanthin (896.51 ± 101.76), and β-carotene (615.61 ± 85.54) were found in the liver, with the eye and plasma following. When comparing the plasma and liver of the *H. pluvialis*-fed group to the *S. platensis* and *B. braunii* ones, the levels of antioxidant enzymes were higher, while the observed lipid peroxidation was limited. These findings suggest that, in comparison to other carotenoids, astaxanthin derived from *H. pluvialis* has superior antioxidant qualities and bioavailability, through the scavenging free hydroxy radicals in living cells. Accordingly, Murthy et al. [37] comparatively evaluated the hepatoprotective effect of the carotenoids extracted from an algal biomass and synthetic β-carotene in vivo. The findings indisputably reveal that *Dunaliella* carotenoids have superior hepatoprotective properties than *Spirulina* carotenoids, due to the combination of carotenes and xanthophylls, while *Spirulina* solely contains β-carotene, according to the high-performance liquid chromatography of the extracts. *Dunaliella*’s increased protection suggests that combined carotenoids have superior biological activity compared to carotene alone. The study’s findings also imply that carotenoids derived from algae have a stronger antihepatotoxic impact than synthetic and naturally occurring β-carotene alone. 

#### 2.1.1. Astaxanthin

**Figure 2:**
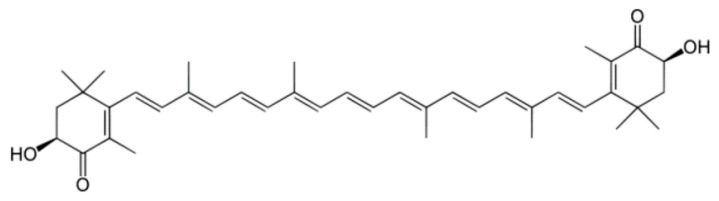
Chemical structure of astaxanthin.

The lipid-soluble, dark-reddish-pigment astaxanthin (3,3-dihydroxy-β, β-1-carotene-4,4′-dione) is a keto-carotenoid (xanthophyll) with strong antioxidant properties. With 100-times higher action than α-tocopherol (Vitamin E), astaxanthin is considered one of the strongest antioxidants discovered to date. Astaxanthin’s unique properties can be attributed to its concluding ring moiety (Figure 2), which enables it to capture free radicals and thus limit lipid peroxidation [38,39]. In biological systems, astaxanthin is mostly found esterified, due to the bonds formed between its hydroxyl group (OH) and fatty acids [38]. The most abundant source of astaxanthin in nature is the microalga *Heamatococcus pluvialis*, containing approximately 5% of its dry weight as astaxanthin, mostly esterified with oleic, palmitic, and linoleic acids [40,41]. However, natural astaxanthin production has a significantly higher cost when compared to synthetic astaxanthin, even reaching 6400 EUR/kg, while synthetic astaxanthin production is priced much lower, at 880 EUR/kg [38,42]. Due to the processes used in its manufacture, synthetic astaxanthin is typically more affordable to produce than natural astaxanthin. Typically, low-cost and widely available petrochemicals or other synthetic precursor compounds are used to produce synthetic astaxanthin chemically, allowing for more control over the concentration and purity. As opposed to this, natural astaxanthin comes from yeast, crustaceans, or microalgae, with the major natural provider being *H. pluvialis* [38,43]. The cost is thus highly affected, as natural astaxanthin production requires significant resources for its cultivation, including land, water, nutrients, and energy. The extraction and purification processes for natural astaxanthin can also be complex and expensive. Over time, the relative costs of manufacturing may be impacted by changes in the market, technology, and regulatory environments. As of today, only natural astaxanthin from *H. pluvialis* has been approved by the FDA and EFSA for human supplementation [44]. 

Astaxanthin has been widely studied by researchers to determine its toxicological safety and bioavailability, antioxidant properties, and health benefits, leading to remarkable findings both in vitro and in vivo. A study of the astaxanthin-enriched fraction of microalga *H. pluvialis*’s acute toxicity, following the treatment of mice and rats with repeated concentrations (500–5000 mg/kg body weight) for 24 h demonstrated no toxicological characteristics and no mortality. Furthermore, the long-term toxicity of the enriched fraction was evaluated for three months, resulting in no discernible variations in blood chemistry markers or histopathological investigations, suggesting that the median lethal dose is up to 5000 mg of the fraction/kg of body weight [45]. The pigment’s safety for consumption during pregnancy was also evaluated, proving to be safe for pregnant mice both in the short term and in the long term, with an oral LD50 of more than 20 g/kg, while keeping intact the chromosomes and mitotic apparatus of the pregnant mice [46].

**Figure 3:**
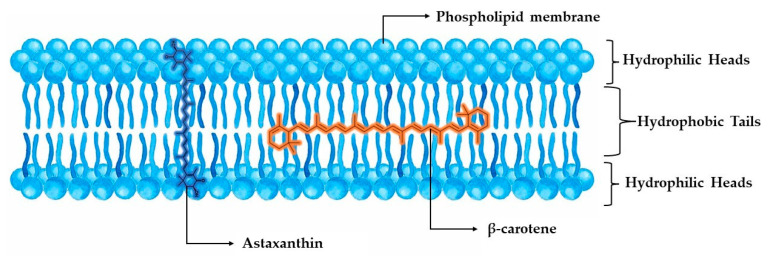
Placement of astaxanthin and β-carotene in phospholipid membrane.

When compared to other carotenoids, astaxanthin has been proven to demonstrate superior antioxidant effects, due to its unique structure and functional reactive end groups. Specifically, Naguib et al. [47] developed a novel fluorometric assay, utilizing BODIPY 665/676 as the indicator, Trolox as the calibrator, and AMVN as the peroxyl radical generator for experimenting with liposomal and organic media, concluding that the relative reactivities of Trolox, astaxanthin, α-tocopherol, β-carotene, lutein, α-carotene, and lycopene were 1.0, 1.3, 0.9, 0.5, 0.4, 0.2, and 0.4, respectively. A more targeted comparison between the most powerful carotenoids, astaxanthin and β-carotene, correspondingly demonstrated astaxanthin’s superiority in liposome peroxidation induced by ADP and Fe^2+^, with a 2-fold potential for inhibiting their production. A remarkable finding is the ability of astaxanthin’s ring moiety to scavenge free radicals both in the membrane and on the membrane’s surface, while both carotenoid polyene chains trapped free radicals only inside the membrane. This antiperoxidative behavior against liposomes can offer insight into the different placement of the molecules inside the cellular phospholipid membrane, where the polar ends of astaxanthin can interact with the hydrophilic region of the membrane by forming hydrogen bonds, while β-carotene only has the ability to intervene in oxidation through the lipophilic part of the membrane (Figure 3) [48]. 

Astaxanthin’s antioxidative effects have been linked to several health benefits by researchers, fortifying the need for carotenoid utilization in the nutraceutical industry. Specifically, an imbalance between the generation and buildup of oxygen-reactive species (ROS) in cells and tissues, in combination with the biological system’s capacity to detoxify these reactive metabolites, results in oxidative stress. When exacerbated by environmental stressors, oxidative stress can lead to severe disease and impair human health irreversibly. Astaxanthin has demonstrated different pathways for battling several types of cardiovascular diseases and cancer, the two major causes of death worldwide [49]. Atherosclerosis and plaque accumulation in the central arteries and vessels of the human body can lead to heart attacks and ischemic episodes [50]. Hypertension, hyperglycemia, hypercholesterolemia, and hypertriglyceridemia are significant risk factors for coronary heart disease (CHD), with inflammation and elevated oxidative stress functioning synergistically for CHD’s prevalence. Supplementation with an astaxanthin-rich *H. pluvialis* extract (0.03% wt) in mice fed a high-fat and -cholesterol diet, effectively altered lipid peroxidation and amplified antioxidant defense mechanisms by adjusting the antioxidant gene expression in the mice’s livers, resulting in lowered cholesterol and chronic inflammation [51]. Astaxanthin’s hypocholesterolemic and hypotriglyceridemic effects have been cross-validated in human trials, where it was concluded that astaxanthin administration for 8 weeks significantly reduced plasma triglycerides, total cholesterol, and low-density lipoprotein (LDL) levels; suppressed inflammatory cytokines production; and mitigated hemostatic disorders in type 2 diabetes patients, thus alleviating the thrombotic risk [52]. Apart from the above, the antidiabetic properties of astaxanthin, according to researchers, include improved glucose metabolism, reduced blood pressure, increased insulin sensitivity, and the promotion of glycemic control in both type 2 diabetes mellitus patients and healthy subjects with prediabetes, after being supplemented for 8 [53] and 12 [54] weeks with daily doses of 8 mg and 12 mg, respectively.

As far as cancer is concerned, astaxanthin has demonstrated great potential for detaining metastasis and even inducing apoptosis of cancer cells. Kurihara et al. [55] determined the immunological value of astaxanthin in conjunction with a restraint stress treatment, and examined the impact of the carotenoid on the antitumor immune activation of natural killer (NK) cells inhibited by stress in mice. Four days of oral astaxanthin (100 mg/kg/day) supplementation improved the immunological dysfunction brought on by chronic stress exposure, by diminishing the hepatic metastasis of mastocytoma P815 tumor cells effectively, especially when compared to α-tocopherol and β-carotene. Palozza et al. [56] examined the potential of *H. pluvialis*, rich in astaxanthin extract (10.2% wt), for inhibiting cancer metastasis in HCT-116 colon cancer cells. *H. pluvialis* extract (5–25 μg/mL) halted the cell cycle progression to suppress cancer cell growth in a dose- and time-dependent manner and, at higher concentrations (15–25 μg/mL), promoted apoptosis by altering the apoptosis-related proteins and kinases signaling. The use of *H. pluvialis* extract in human nutrition is fully advocated by Palozza et al. due to their observation that its impacts on cell proliferation and apoptosis were more prominent than those of purified astaxanthin at the same concentration [56].

Astaxanthin’s antioxidant effects have also been manifested through neuroprotective, photoprotective, and anti-inflammatory responses in humans. Park’s study of the action of dietary astaxanthin supplementation for 8 weeks in young healthy females demonstrated that astaxanthin improved the immune response and reduced inflammation and the DNA oxidative damage biomarker C-reactive protein [57]. Moreover, inflammation and elevated cytokines production have been linked with depression and neurοdegeneration. When depression symptoms were stimulated in rodents by lipopolysaccharide administration, trans astaxanthin was able to ameliorate the depressive-like behavior of LPS-induced depression mice, by reducing the pro-inflammatory cytokine production through the nuclear factor kappa B (NF-κB) pathway, thus suggesting its potential use in clinical therapy for major depressive disorders [58]. Nakajima et al. [59] demonstrated astaxanthin’s neuroprotective effects on retina ganglion cells both in vitro and in vivo by neutralizing hydroxyl radicals (OHs), superoxide anion (O_2_^−^s), and hydrogen peroxide (H_2_O_2_), ultimately restraining lipid peroxidation and DNA damage. Supplementation with 4 mg astaxanthin/day for 9 weeks in subjects aged 30–60 with sustained UVA skin damage demonstrated protective and age-reversing effects, improving skin texture and appearance [60].

#### 2.1.2. β-Carotene

**Figure 4:**
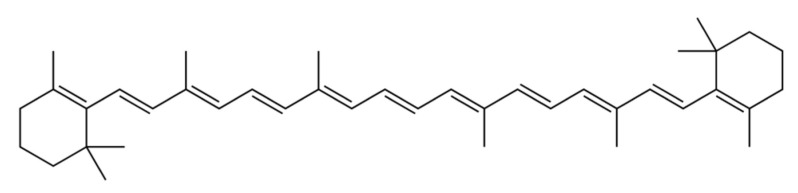
Chemical structure of β-carotene.

**Figure 5:**
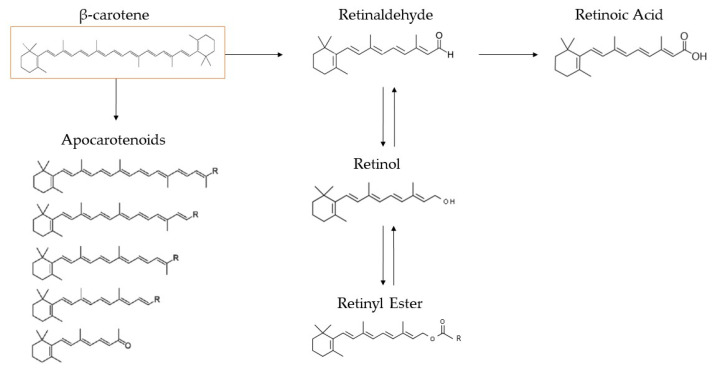
β-carotene’s enzymic metabolism pathway in mammals, as proposed by von Lintig [66].

β-carotene is a hydrocarbon carotenoid (Figure 4) that can be transformed into Vitamin A and related retinoids in mammal organisms, accounting for 30% of dietary Vitamin A consumption in Western civilization [61]. The β-carotene market is estimated to be valued at USD 623.25 million in 2024 and USD 744.89 million by 2029, with a compound annual growth rate of 3.63% throughout the projected period (2024–2029) [62]. β-carotene’s transformation into retinoids is briefly described in Figure 5. The most significant microalga source of β-carotene is *D. salina*, accumulating up to 10–13% dw, in total, of the carotenoid by the proper cultivation stressors [63], with the *Spirulina* strains following, making up 69.5–80% of the total carotenoid content. [30,31]. Furthermore, the profiles of β-carotene acquired from microalgae and chemically generated sources are not the same. Only the (all-E)-isomer is present in synthesized β-carotene, whereas *D. salina*’s β-carotene mostly comprises three isomers: (all-E)-β-carotene (42%), (9Z)-β-carotene (41%), (15Z)-β-carotene (~10%), and other isomers (6%). Given that (9Z)-β-carotene is crucial for antioxidation, microalgae may indeed be capable of delivering natural β-carotene at a relatively low cost and with greater safety, which would enhance its biofunctions [64,65].

Different clinical trials have pinpointed β-carotene’s antioxidant effects and its consequent results in cardiovascular diseases. Three different clinical trials among Finnish and Japanese populations have revealed the negative correlation between serum β-carotene concentration and the risk of cardiovascular disease mortality (including heart disease and stroke), the CRP inflammation biomarker, and sudden cardiac death, especially for male smokers [67,68,69]. Specifically, Karppi et al. [67] demonstrated a 2-fold higher risk of cardiovascular disease mortality for the last quartile of β-carotene serum concentrations. 

*D. salina*’s antioxidant properties have been mainly attributed to its high content of β-carotene. Hu et al. [36] developed an efficient separation technique using a basic HPLC approach to identify the different carotenoids included in microalgae. The quantity of seven pigments in the algal extract that could be separated concurrently in 30 min, according to the results, was 290.77 mg/g of algae, with 92% of them being cis and trans isomers of β-carotene, while the extract’s antioxidant capacity was superior to that of the separate carotenoids in a Trolox equivalent antioxidant capacity (TEAC) assay, reducing the power of the 2,2-diphenyl-2-picrylhydrazyl hydrate (DPPH) radical scavenging assay that was implemented. *D. salina*’s crude hexane extract, rich in β-carotene (0.813%) was tested for its short-term and long-term toxicity by administering 500–5000 mg/kg and 500 mg/kg/day for 3 months to rats, respectively. No significant changes were observed in the hematological parameters, liver enzymes, or histological examinations in either case, thus suggesting that the LD50 of a *D. salina* rich in β-carotene extract is up to 5000 mg/kg and is generally safe for long-term consumption at doses of 500 mg/kg/day [70].

β-carotene exhibits high antioxidant activity that can be later perceived as having anti-inflammatory effects. Lin et al. [71] assessed *D. salina*’s extract content of β-carotene and its antioxidant and anti-inflammatory potential against murine macrophage (RAW264.7) cells with a pseudorabies virus (PRV), induced as a response to oxidative stress. The microalga was found to contain 91.8% β-carotene, while it utterly prevented ROS generation at doses of 50 mM and 100 mM, displaying high antioxidant action. At the same time, it inhibited virus replication by preventing pro-inflammatory interleukin accumulation via the NF-κΒ signaling pathway. In vivo, *D. salina* demonstrated those assets in a hepatoprotective manner. More specifically, Murthy et al. [72] evaluated *D. salina* powder’s extract, with a relative percentage of 86.5% β-carotene, for its competency against oxidative stress induced in rats by CCl_4_ and compared it to the effect of synthetic β-carotene under the same conditions. The group treated with the extract demonstrated a 75.0% restoration in peroxidation, with a great degree of antioxidant hepatic enzymes’ function, while the group treated with synthetic pigment only restored their liver lipid peroxidation by 23.0%, revealing the natural carotene’s dominance over the synthetic one. Similar conclusions were reached by Hsu et al. [73] in corresponding research involving *D. salina* administration to mice with CCl4-induced liver damage twice a week for 4 weeks in total. The alga supplementation reduced lipid peroxidation, serum triglycerides, and cholesterol, and increased hepatic enzyme activity, while liver damage was significantly diminished.

The alga’s activity against UV radiation was also assessed with remarkable findings concerning its photoprotective effects. Specifically, in a placebo-controlled study, the administration of 24 mg/day of naturally derived β-carotene for 12 weeks demonstrated equal effectiveness against UV-induced erythema compared to supplementation with 24 mg/day of three different carotenoids (β-carotene, lutein, and lycopene, 8 mg each), highlighting β-carotene’s properties [74]. Accordingly, Tsai et al. [75] showed that *D. salina* administration for eight days to mice with sustained corneal oxidative damage caused by UVB radiation significantly ameliorated the injury and increased antioxidative enzyme activity in the area, resulting in lessened lipid peroxidation.

#### 2.1.3. Lutein and Zeaxanthin

**Figure 6:**
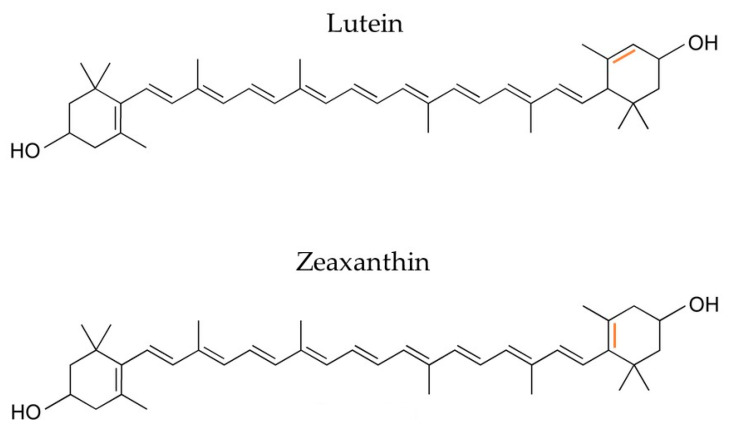
Lutein and zeaxanthin only differ in a double bond placement.

Lutein is a non-provitamin A, xanthophyll carotenoid with an expected market value of EUR 409 million with a CAGR of 6.10% over the projected time frame (2020–2027) [76]. It is abundant in green vegetables and herbs, as well as in microalgal strains, with the most important being *Chlorella pyrenoidosa*, *zofingiensis*, and *Spirulina platensis*. Its isomer compound, zeaxanthin, only differs at the position of one of the ring’s double bonds (Figure 6), with similar natural sources and a much smaller market size estimated at USD 210 million with a CAGR of 8.2% until 2030 [76]. Both molecules are key antioxidant elements of the human retina, with a ratio of lutein/zeaxanthin of 3:1 [77], inhibiting ophthalmological diseases caused by light exposure. 

Lutein and zeaxanthin have demonstrated several health benefits both individually and synergistically. Kim et al. [78] exhibited its atherosclerosis prevention effects by administering a 0.1 g lutein/100 g diet to guinea pigs fed a high-cholesterol diet (0.25 g/100 g diet), observing reduced inflammatory aortic cytokines and middle-sized LDL, the diminution of atherogenic lipoproteins in circulation, and narrowed aortic plaque. Quiao et al. [79] displayed its potential protective role against monosodium iodoacetate (MIA)-induced osteoarthritis in primary chondrocyte cells, where lutein enhanced chondrocyte cell viability and delivered substantial cytoprotection by augmenting the antioxidant defense systems and minimizing oxidative stress. By downregulating pro-inflammatory cytokines and inflammatory proteins, lutein supplementation demonstrated anti-inflammatory benefits and preserved the capability of the mitochondrial membrane to lessen MIA-induced apoptosis. In a randomized, double-blind, placebo-controlled study, 10 mg/day lutein administration for a year improved the macular pigment optical density in patients with early age-related macular degeneration (AMD), enhancing their total visual acuity [80]. On the other hand, zeaxanthin ester isolated from *D. Salina* ameliorated cardiac dysfunction when given for 28 days to rats with d-galactose-induced cardiac dysfunction in therapeutic doses of 250 µg/kg, while reversing age-related symptoms, including ECG patterns, histopathological cardiac tissue lesions, hepatic biomarkers increase, and inflammatory cytokines production, by stimulating retinoid receptors in the cardiac tissues [81].

The combination of zeaxanthin and lutein exhibits antidiabetic, cardioprotective, and cognitive-enhancement effects. Qi et al. extracted the two isomers from dry *Chlorella ellipsoidea* (140 mg), and demonstrated their ability to inhibit a-glucosidase action from mammalian (rats), bacterial (*B. stearothermophilus*), and yeast (*S. cerevisiae*) sources, thus being an excellent candidate for supplementary diabetes mellitus treatment [82]. Chung et al. [83] compared different carotenoids’ anti-inflammatory responses in patients with acute coronary syndrome and stable angina. Only lutein and zeaxanthin were negatively associated with interleukin production, even 3 months after the completion of the study, thus offering atheroprotection.

### 2.2. Proteins and Bioactive Hydrolysates

According to the UN, the global population is expected to surpass nine billion by 2050 [84] and, in combination with the steady improvement in living and nutritional standards, the demand for protein is expected to rise by as much as 78% [85]. This upsurge is due to the fact that proteins are considered the most significant component in terms of providing sufficient nourishment [85]. However, conventional protein resources, including meat, eggs, and dairy, are traditionally derived from livestock production, an agricultural sector that strains the environment by limiting land biodiversity and accelerating the nitrogen cycle, producing 12% of the emitted greenhouse gases [86]. To ensure food security while utilizing the limited available land and water resources, it is vital to explore alternative and sustainable protein sources from natural habitats. Microalgae are considered an excellent candidate for protein accumulation due to their high content of bioactive proteins (Table 2), with high nutritional and medicinal values due to the structure of the contained amino acids (Table 3), which display unique properties when forming peptides. Additionally, their protein content can be maximized by manipulating the cultivation stressors that act as growth parameters, including light, pH, and nutritional medium contents. 

This review will focus on phycobiliproteins—protein complexes unique to algae—phycocyanin and phycoerythrin, as well as the bioactive peptides derived by hydrolyzing algal biomasses.

#### 2.2.1. Phycobiliproteins 

Phycobiliproteins (PBPs) are a class of complex proteins that serve as auxiliary pigments in the algal chloroplast, by harvesting light in the visible spectrum and transferring the stored energy to chlorophyll molecules indirectly in a sufficient degree (95%), while preventing photolysis of the microalgal cells occurring during photosynthesis [95,96]. Specifically, depending on the wavelength absorbance, they can be classified as (i) purple phycoerythrins, λ_max_ = 540–570 nm (ii); orange phycoerythrocyanins, λ_max_ = 560–600 nm; (iii) blue phycocyanins, λ_max_ = 610–620 nm; and (iv) blue-green allophycocyanins, λ_max_ = 650–655 nm [96]. They are biodegradable, innocuous, hydrophilic compounds, of a great molecular weight (220–300 kDa), composed of two main subunits, α and β, which stabilize each other through electrostatic forces and form steady trimers (αβ)_3_ or hexamers that promote solar light absorbance [97]. Their chromophore parts consist of open chain tetrapyrroles bonded to cysteine covalently via thioether linkages, which are different for every subtype of PBP, functioning as a diagnostic for the integrity of its structure, since it maintains its color only in its natural form [98].

PBPs are mostly found in cyanobacteria (50% of their total protein), rhodophytes, cryptomonads, and cyanelles, and are considered highly bioactive compounds according to the research, with great market potential, with their price ranging from USD 5000–33,000/g [99].

##### Phycocyanin

**Figure 7:**
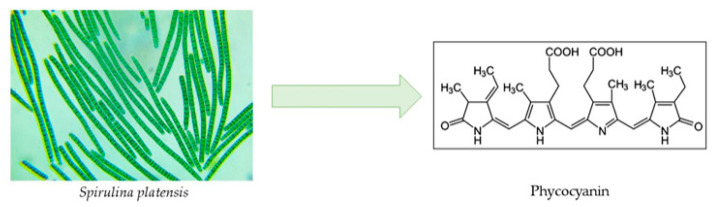
Chemical structure of phycocyanin.

Nearly all phycobiliprotein-containing organisms, such as cyanobacteria, red algae, glaucophytes, and certain cryptophytes, contain phycocyanins (PCs) (Figure 7). Three kinds of PCs are distinguished based on their spectral characteristics: (i) cyanobacteria are the only organisms that contain C-PC (λ_max_~615–620 nm), (ii) phycoerythrocyanin (PEC, λ_max_~575 nm) is present in only certain cyanobacteria, and (iii) R-PC is primarily present in red algae (λ_max_~615 nm) [100]. PC’s several health benefits—which will be discussed—have rendered the compound of great value for the nutraceutical industry, with a projected market value as high as USD 245.5 million through 2027 [101]. The most researched subtype is C-PC, derived from *Spirulina platensis* cyanobacterium, accounting for up to 29% of the dry biomass [102]. 

PC has proven to be a molecule with great antioxidative and anti-inflammatory properties. Grover et al. [103] evaluated C-PC’s safety, antioxidant, and immunomodulatory effects in vivo, ascertaining its action in a dose-dependent manner without showing toxicity signs at more than 2000 mg/kg bw in mice, while its radical scavenging activity and antioxidative enzyme activity preservation was equivalent to that of Vitamin E. C-PC’s anti-inflammatory action has been linked to histamine suppression, with a remarkable attenuation of carrageenan-evoked thermal hyperalgesia [104].

The antioxidant potential of PC is manifested in different manners, suggesting protective effects against radiation, cardiovascular risk factors, and neurodegenerative diseases. Oxidative stress caused by UVB radiation highly scars the outer skin layers, even resulting in melanoma and other types of skin cancers [105]. Phycocyanin’s photoprotective effects can attenuate such outcomes. Specifically, *Spirulina*-derived C-PC-enhanced keratinocyte cells’ viability when treated with UVB radiation increased by 29.5%, while ROS accumulation was limited by half after treatment, and was even able to restore the skin’s natural barrier against radiation [106,107].

On the other hand, C-PC derived from *Spirulina* species has been proven to be highly effective in treating CVD symptoms, demonstrating a hypocholesterolemic effect in rats, superior to that of *Spirulina* concentrated extract or casein, with the key difference being its higher content of cystine and glycine [108]. Its cholesterol-lowering effect is a major factor in the prevention of atherosclerosis, as suggested by Riss et al. [109] after administering PC in doses of 7.4 mL/kg/day to hamsters fed an atherogenic diet for 12 weeks, concluding that PC supplementation limited the oxidative stress markers indicative of lipid deposition in the aorta. Apart from its hypocholesterolemic effect, C-PC has been proposed as possessing strong antithrombotic potential, even in nanomolecular concentrations (0.5–10 nM), against platelet aggregation, limiting the phenomenon by up to 92% [110]. 

As far as neurodegenerative diseases are concerned, C-PC has successfully limited symptoms of Alzheimer’s disease (AD) and multiple sclerosis (MS). The behavioral and cholinergic activity of rats with cognitive dysfunction similar to AD were evaluated after injection with 100 mg PC/kg for 28 days, a dose proven to be effective against hippocampal neuroinflammation, improving the cognitive skills of the rats [111]. Similar results were obtained by Li et al. [112] after treating mice with cognitive dysfunction induced by amyloid-beta (Aβ) plaque with pure PC from *Spirulina platensis*, leading to the restoration of partial memory performance, and accompanied by a reduction in inflammatory cytokines production. Moreover, Cervantes-Llantos et al. [113] provided information on the potential use of C-PC and its prosthetic group phycocyanobilin for battling MS. Specifically, oral administration of an *S. platensis* extract-enriched in C-PC (30%) at a concentration of 200 mg C-PC/kg for 15 days significantly attenuated the progression of the disease, while limiting the characteristic demyelination and inflammation phenomena occurring in the rats’ brains.

When studied as a potential anticancer agent, PC has demonstrated noteworthy properties for battling several types of the disease. Hao et al. [114] examined the in vitro anti-tumor activity of phycocyanin purified from *Spirulina* against non-small-cell lung cancer (NSCLC) cells. The findings demonstrated that by influencing multiple genes, phycocyanin dramatically triggered apoptosis and cell cycle arrest in addition to inhibiting the NSCLC cells’ potential to migrate, proliferate, and form colonies. It has also been reported that PC exhibits anticancer potential when supplemented in doses of 100 mg/kg in mice with colitis-associated colorectal cancer, by protecting the gut microbiota, suppressing tumor proliferation, and limiting inflammatory cytokines production through manipulating gene expression [115]. PC was effective even against pancreatic adenocarcinoma, one of the types of cancer that is the most lethal and nonresponsive to conventional chemotherapeutic drugs, as reported by Liao et al. [116]. Specifically, PC induced apoptosis and autophagy of pancreatic cancer cells, both in vitro and vivo, while keeping normal cells intact. Another type of cancer that is nonresponsive to chemotherapy is triple-negative breast cancer, against which C-PC obtained from *S. platensis* was proven to be effective in vitro, by inhibiting the reproduction and metastasis of the tumor cells, while promoting apoptosis via the MAPK signaling pathway [117]. 

Several researchers have highlighted PC’s capacity to battle diabetes mellitus symptoms successfully. A molecular docking analysis of the pigment demonstrated inhibitory activities against α-amylase and α-glucosidase enzymes, by attaching itself to the catalytic site and interfering with the binding of the substrate to the enzyme. An in vitro evaluation of the activity revealed that 1000 ppm of PC inhibited human salivary α-amylase activity by 51.13% [118]. In vivo studies of PC administration in diabetic mice confirmed its antidiabetic effect. Ou et al. [119] treated alloxan-injured diabetic mice with 100 or 200 mg PC from *S. platensis*/kg/day for four weeks, which led to a simultaneous increase in blood insulin and a decrease in blood glucose levels, suggesting a novel antidiabetic effect. In compliance with these results, oral supplementation of 100 mg PC/kg, once per day for 3 weeks, improved insulin sensitivity and plasma secretion, and lowered the total cholesterol, glucose, and triglyceride levels in the blood and liver of spontaneously diabetic mice suffering from obesity, hyperglycemia, and hyperinsulinemia. Ultimately, PC controls the metabolism of glucolipids and could be a strong candidate for treating type 2 diabetes [120]. 

Finally, PC has demonstrated unique antimicrobial and antiviral properties. Specifically, through interactions based on in silico molecular modeling, strong correlations between C-PC and HIV-1 proteins have been discovered, while in vitro mechanistic research has verified its selectivity, by inhibiting reverse transcriptase and protease enzymes. Notably, concentrations of 0.3566 mg PC/mL successfully inhibited HIV-1 replication by 80%, while being safe for normal cells at doses up to 0.5 mg/mL [121]. As far as bacteria are concerned, C-PC strongly suppresses the action of several resistant strains in a dose-dependent manner, as demonstrated in Table 4. It should be pointed out that the larger the inhibition zone of the microbe, the higher the antimicrobial potency of the agent. These results are highly suggestive of PC’s use in the nutraceutical industry for cosmetics formulation and medical applications.

##### Phycoerythrin

**Figure 8:**
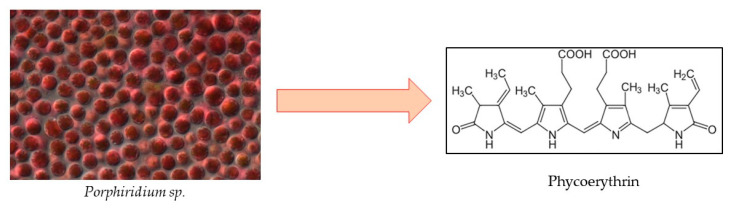
Chemical structure of phycoerythrin.

Even though research has mainly focused on PC due to its abundance and many health benefits, a fellow pigment, phycoerythrin (PE) (Figure 8), has shown great potential as a bioactive compound derived from microalgae. Red algae, particularly *Porphyridium* sp., have been extensively researched and used for R-PE on a commercial basis for a long time, rendering them the primary source of PE. Cyanobacteria, however, may develop into a different source for C-PE accumulation [125]. The PE market was valued at USD 4.2 million as of 2022, and is expected to reach USD 7.6 million by 2032, with a CAGR of 6.2% [126].

PE has been demonstrated to possess antioxidant, anti-aging, antidiabetic, and neuroprotective effects. PE purified from the cyanobacterium *Halomicronema* sp. R31DM has exhibited antioxidant effects in a dose-dependent fashion both in vitro, by DPPH radical scavenging activity (DPPH), a ferric ion-reducing ability of plasma (FRAP) assay, and a reducing power (RP) assay, and in vivo when administered to *C. elegans*, a eukaryotic model with aging mechanisms similar to that of humans. The increased survival rates of the groups fed with higher doses were attributed to PE’s structure and specifically positive contributing amino acid residues [127]. Soni et al. [128] revealed C-PE’s potency against oxidative stress by administering 25 or 50 mg C-PE purified from *Phormidium tenue* microalga/kg bw/day for 28 days to rats with induced diabetes, observing a decline in all diabetes-related biomarkers, such as glucose levels in the blood, cholesterol, and triacylglycerol, phenomena attributed to the deceleration of the oxidation rate. When tested as a therapeutic agent against neurodegenerative diseases, C-PE was effective at fighting Alzheimer’s disease (AD) symptoms, such as muscle paralysis, in a dose-dependent manner, after supplementation with *C. elegans*, via an interaction with the beta-site amyloid precursor protein cleaving enzyme-1 (BACE1) [129]. Moreover, C-PE purified from the cyanobacterium *Lyngbya* sp. A09DM increased the life span of *C. elegans* up to 41.6 ± 2.5% under thermo-stress, and up to 63.1 ± 6.4% under oxidative stress, while limiting the action of the human amyloid-beta peptide, a factor with a central role in the pathology of AD. It should be highlighted that in vitro studies have demonstrated PE’s greater antioxidant effect when compared to PC and allophycocyanin [130,131].

#### 2.2.2. Bioactive Hydrolysates 

Protein hydrolysates and their purified peptides (amino acid consequences) have emerged as novel bioactive compounds with antioxidant, anticancer, and antihypertensive activities, amongst others. They can be obtained through enzymatic hydrolysis, exerting physiological effects that can only be exploited after treatment. The peptide action is highly influenced by the amino acid sequence and composition, which is usually defined by the 3–20 contained amino acids [132,133]. Microalgae peptides can be efficiently derived from protein-rich strains (Table 2), even as a byproduct after the major accumulation of other bioactive compounds, creating an opportunity for the development of a sustainable industry based on a circular economy [134,135]. 

Table 5 highlights the great antioxidant capacity in vitro of different microalgal strains. 

The pepsin-hydrolyzed peptide from *C. vulgaris* waste, obtained as a byproduct rich in protein after algae essence manufacturing, was shown to be powerful, especially for scavenging peroxyl radicals and, thus, protecting cells against DNA damage, with an observed activity 26-fold stronger than Trolox, and with superior antioxidative properties when compared to the common antioxidants ascorbic acid and butylated hydroxytoluene [135,137]. Similarly, Ko et al. [136] demonstrated that when *C. ellipsoidea* biomass was hydrolyzed by pepsin, a bioactive peptide was obtained that increased cell survival rates in a dose-dependent manner, reaching 79.4% for a concentration of 100 μM, protecting cells from free radical oxidative stress. The peptide obtained from *I. zhanjiangensis* by mimicking gastrointestinal digestion sufficiently scavenged ROS produced in liver cells by alcohol consumption. The antioxidant and hepatoprotective effects were attributed mainly to the hydrophobic and cyclic amino acid residues, constituting 59.98% and 13.95% of the total amino acids, respectively. The hydrophobicity of the amino acid composites as an indication of antioxidant activity was also highlighted by Bai et al. [134] after isolating 230 peptides from *A. maxima* biorefinery residues by an alcalase treatment, suggesting that the increased lipid solubility benefits intracellular interactions with radicals. However, it was noted that certain biopeptides are highly susceptible to degradation in the gastrointestinal tract, thus demanding stability enhancement and controlled release, attributes that could be obtained through encapsulation in proper matrices. 

Microalgae bioactive peptides have been designated as CVD preventive factors, with their main applications in hypertension and atherosclerosis treatment. Angiotensin-converting enzyme (ACE) inhibitors have been thoroughly researched and are the go-to treatment option for blood pressure control [140]. The Thr-Met-Glu-Pro-Gly-Lys-Pro peptide derived from *Spirulina* after in vitro gastrointestinal digestion non-competitively bound with ACE, inhibiting the enzyme’s action through stabilization with hydrogen bonds and Van der Waals interactions, finally reducing ROS production in angiotensin II-stimulated endothelial cells [141]. In vivo testing of the ACE inhibitory action of the peptidic fractions of *C. vulgaris* and *S. platensis* at a dose of 200 mg/kg bw to spontaneously hypertensive rats reduced their blood pressure for up to 4 h after administration in a comparable manner to captopril, an antihypertensive drug. With further purification, the following peptides were identified: Ile-Val-Val-Glu (inhibitory against ACE with an IC50 of 315.3 µM), Ala-Phe-Leu (63.8 µM), Phe-Ala-Leu (26.3 µM), Ala-Glu-Leu (57.1 µM), and Val-Val-Pro-Pro-Ala (79.5 µM) from *C. vulgaris*; Ile-Ala-Glu (34.7 µM), Phe-Ala-Leu, Ala-Glu-Leu, Ile-Ala-Pro-Gly (11.4 µM), and Val-Ala-Phe (35.8 µM) from *S. platensis*. It should be recognized that almost every peptide contains residues of the amino acids phenylalanine, glutamic acid, and proline, whereas the most drastic ones have proline, phenylalanine, or tyrosine at the carboxy end and valine and isoleucine at the amino end, characteristics that might be responsible for the ACE inhibitory effect [142]. Identical results were extracted by Ko et al. [143] regarding the tetrapeptide Val–Glu–Gly–Tyr isolated from *C. ellipsoidea* with alcalase –proteolytic hydrolysis, which was proved to be stable against gastrointestinal tract enzymes. Samarakoon et al. [144] exploited *Nannochloropsis oculata* biomass and identified the novel peptides Gly-Met-Asn-Asn-Leu-Thr-Pro and Leu-Glu-Gln, which exhibited significant ACE inhibitory activity with IC_50_ values equal to 123 μM and 173 μM, respectively, while their action was attributed to their contents of proline, leucine, methionine, glutamic acid, and glycine. The pepsin-hydrolyzed fractions were nontoxic towards human umbilical vein endothelial cells. 

It has been demonstrated that the formation of clinically discernible atherosclerotic plaques in the coronary arteries precedes endothelial dysfunction/activation, which is currently recognized as an early critical phase in atherogenesis [145]. Histamine-induced endothelial cell activation has been suppressed by peptides Leu-Asp-Ala-Val-Asn-Arg and Met-Met-Leu-Asp-Phe, which were obtained from *S. maxima*’s in vitro gastrointestinal hydrolysis. By preventing the synthesis of adhesion molecules and restricting monocyte adhesion and migration onto activated endothelial cells, the peptides mentioned above have been proposed as therapeutics for the treatment of cardiovascular diseases like atherosclerosis [145]. The same conclusions were reached for the *Chlorella*-11 peptide (Val-Glu-Cys-Tyr-Gly-Pro-Asn-Arg-Pro-Gln-Phe) accumulated from *C. ellipsoidea* algal waste, which has demonstrated promising results as an antiatherosclerotic agent, with the additional advantage of limiting endothelial cell permeability [146].

Protein hydrolysates have also been researched for the identification of bioactive peptides with anticancer properties. The peptide obtained from *Chlorella vulgaris* waste using pepsin demonstrated antiproliferative effects against gastric cancer cells while keeping normal human lung cells intact, by inducing cell apoptosis and limiting cancer cell growth [135]. Wang et al. [147] applied a three-step hydrolysis technique (using pepsin, trypsin, and chymotrypsin enzymes) to the proteins extracted from *S. platensis*. The most active peptidic fraction, at a concentration equal to 500 μg/mL, showed antiproliferative effects against five tumor cells: liver cancer, breast cancer, gastric cancer, lung cancer, and colon cancer, with inhibition degrees greater than 80%. It should be highlighted that for liver and breast cancer, the inhibitory percentage was greater than that of the conventional chemotherapy drug 5-FU. A special peptide was identified, with the sequence His-Val-Leu-Ser-Arg-Ala-Pro-Arg, which exhibited a statistically substantial degree of inhibitory action (IC_50_ value of 99.88 μg/mL) on colon cancer cells, but only a small percentage (5.37% at 500 μg/mL) on normal liver cells, suggesting that the peptide was selective for cancerous cells and not healthy ones. In a study implemented by the same researchers using *S. platensis* as a source for bioactive hydrolysates, the peptidic sequence Tyr-Gly-Phe-Val-Met-Pro-Arg-Ser-Gly-Leu-Trp-Phe-Arg displayed antiproliferative effects against all five cancer cells, with the maximum activity observed against lung cancer cells. Similarly, the peptide was characterized as safe for normal liver cells, thus displaying selectivity [148].

A field of great research interest has been the immunostimulant and anti-inflammatory activities of bioactive microalgae peptides. The administration of 500 mg/kg of *C. vulgaris* enzymatic protein hydrolysate for 8 days to undernourished mice boosted their immune system by activating the phagocytes, with an evident increase in leukocyte counts in the peripheral blood of 128% [149]. Vo et al. [150] identified the peptides Leu-Asp-Ala-Val-Asn-Arg and Met-Met-Leu-Asp-Phe from S. maxima gastrointestinal hydrolysis and uncovered their inhibitory effects against histamine and ROS release and production from antigen-stimulated mast cells in a dose-dependent manner, thus limiting the inflammatory reaction. The same bioactive peptides also suppressed allergic reactions to FcRI-mediated mast cells, while limiting cytokine production [151]. The antiatherosclerotic peptide *Chlorella*-11 mentioned before, also demonstrated anti-inflammatory activity in a dose- and time-dependent manner both in vitro and in vivo, as it attenuated the inflammation progression caused by lipopolysaccharide-activated macrophage cells and thermally injured rats, respectively [152].

### 2.3. Polyunsaturated Fatty Acids 

**Figure 9:**
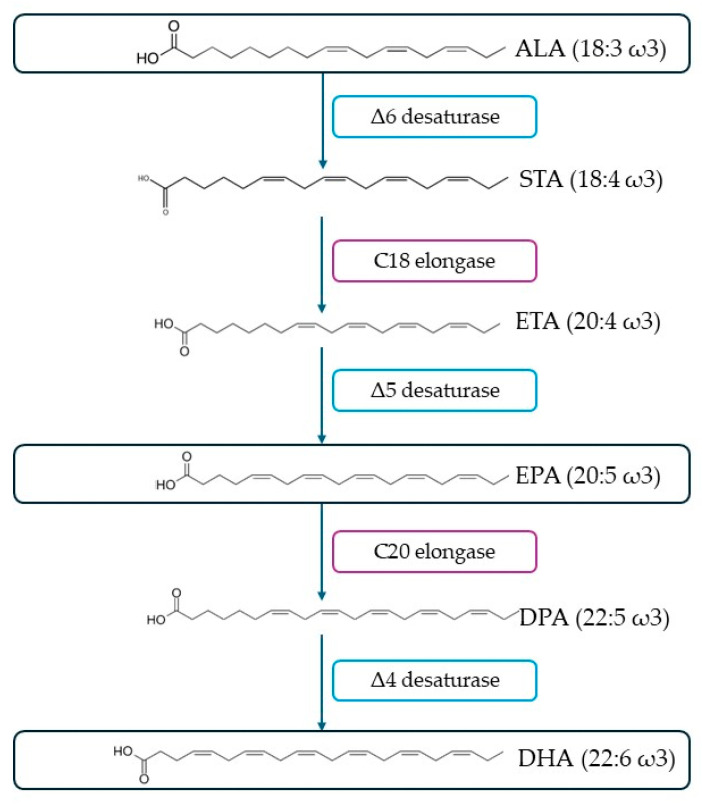
Conversion of ALA to EPA and DHA through enzymatic mechanism, as proposed by Doughman et al. [158].

Fatty acids are the fundamental building blocks of lipids, which serve an important role as biological agents in processes involving energy storage, cell membrane structure, and fluidity, as well as signaling pathways [153,154]. Structurally, fatty acids are composed of a hydrocarbon skeleton equipped with a carboxylic end group [153]. Depending on their saturation degree, they can be categorized into saturated (no double bonds), monounsaturated (MUFAs) (one double bond), or polyunsaturated fatty acids (PUFAs) (two or more double bonds). Of these categories, PUFAs are of the greatest importance due to their participation in the different biological systems in humans. They can be further divided into ω-3 and ω-6 depending on the first carbon of the first double bond from the methyl end. ω-3 and ω-6 PUFAs are considered essential for humans, since they cannot be biosynthesized by them, due to the lack of enzymes Δ12- and Δ15- desaturases, which are responsible for introducing double bonds into the ω-6 and ω-3 carbons, respectively, but, at the same time, are necessary for their diet [155,156]. Linoleic acid (LA, 18:2ω6) and α-linolenic acid (ALA, 18:3ω3) are considered the precursors of many PUFAs, such as docosahexaenoic acid (DHA, 22:6ω3) and eicosapentaenoic acid (EPA, 20:5ω3), which are produced from ALA (Figure 9). However, their bioconversion rate is low, ranging between 10 and14%, and thus insufficient to cover the nutritional needs of humans [157]. Subsequently, they should be provided directly through dietary sources. 

Traditionally, EPA and DHA have been acquired through fatty fish, such as salmon, tuna, herring, mackerel, sardines, and anchovies [159]. Edible oils, such as rapeseed and soybean oil, also contain decent amounts of ω-3 PUFAs [156]. The European Food Safety Authorization has recommended a daily intake of 250 mg EPA + DHA for adults to ensure adequate absorption, a dose corresponding to 2–3 fish servings per week [156,159]. However, the exponential population growth has substantially increased the demand for fish oil rich in ω-3 PUFAs, a reserve that should be distributed between human consumption and aquaculture, as it paradoxically serves as a major component of fish feed by which to increase the farmed fish contents of EPA and DHA [160]. Concurrently, fish are regarded with suspicion, due to the possibility of containing toxins, mercury, and polychlorinated biphenyls (PCBs), which are considered harmful to human health [161], while more and more people worldwide are following vegetarian and vegan diets due to moral and environmental concerns, and the possible health benefits of a diet free of animal products [162]. Even without personal beliefs and preferences in mind, global warming is limiting the EPA and DHA fish products due to rising water temperatures [163].

Undoubtedly, there is an evident need for a sustainable and robust source of ω-3 PUFAs. Microalgae could play a critical role as an EPA and DHA provider, due to their naturally occurring high contents of fatty acids. Table 6 presents the most well-known microalgae species and their fatty acid contents. 

Fish oil has been widely accepted as an efficient source of PUFAs linked to enhanced cardiovascular, mental, and brain health, as well as cancer, diabetes, and inflammation prevention [172]. Research has provided adequate information to consider microalgal oil an excellent substitute with similar functional properties. Van Beelen et al. [173] compared microalgal oil to fish oil as supplements for preneoplastic lesion prevention in rats with azoxymethane (AOM)-induced colonic aberrant crypt foci. Treatment with oils of similar PUFAs content (41% and 34%, respectively, for microalgal and fish) demonstrated an equal activity against early signs of colon carcinogenesis. Correspondingly, Ryckebosch et al. [174] addressed the issue of the feasible consumption of microalgal oils, when comparing the lipid extracts of several microalgae. Amongst them, the highest lipid content was accumulated from *N. oculata*, *N.gaditana*, and *P. lutheri*, accounting almost for 30% of their total biomass content, with the main sources of EPA and DHA being *N. oculata*, *N gaditana*, and *P. tricornutum* (EPA content > 100 mg/g oil), as well as *I. galbana* and *P. lutheri* (DHA content > 40 mg/g oil). In order to fulfill the recommended consumption of 250 mg EPA + DHA/day, extracts from *N. gaditana*, *N. oculata*, *P. lutheri*, *P. tricornutum*, and *T. pseudonana* required doses lower than 2.5 g/day, rendering them proper candidates for fish oil substitution (needed dose: 0.8 g/day). However, due to the richer nutritional profile of the microalgal extracts, which contained decent amounts of carotenoids when compared to fish oil, the first displayed a 3–4-fold antioxidant capacity, revealing supported antiperoxidation protection. 

Microalgal oils have also been found to be effective at supplying the essential PUFAs to people with dietary habits that limit fish product consumption. Geppert et al. [175] studied supplementation with DHA-rich oil from the microalga *Ulkenia* sp. corresponding to 0.9 g DHA/day for 8 weeks to vegetarians, observing a significant increase in the EPA and DHA content of blood cells and plasma phospholipids, while the ω-3 index, a metric of overall health and risk of CVD, remarkably rose from 4.8 ± 0.2 wt% to 8.4 ± 0.2 wt% after 8 weeks, reaching the recommended value spectrum (>8%) [176]. A crossover, randomized controlled trial conducted by García-Maldonado et al. [177] found that daily supplementation with 250 mg DHA/day derived from *Schizochytrium* sp. sufficiently increased circulating n-3 PUFAs concentrations in adults with no dietary restrictions, in vegetarians consuming eggs and dairy, as well as in vegans, with the relative difference being higher for the latter two. 

When studied independently, microalgal oil has been revealed to possess several health effects, similar to those recorded for fish oil. Specifically, microalgal PUFAs have demonstrated robust neuroprotective and antidegenerative effects. *C. sorokiana*, a lipid extract rich in PUFAs (286.7 mg/g), via acute oral administration (30 mg extract/kg rat diluted in 1 mL of sunflower oil) to rats resulted in apparent cognition enhancement and short-term memory improvement, accompanied by an increase in noradrenaline and serotonin in the hippocampus of the rats’ brains [178]. Lai [179] suggested that EPA and DHA from *N. oceanica* cultivated under low-urea conditions, in order to enhance PUFAs production, significantly decreased the damage induced in neuro-2A cells by amyloid-beta protein, which is mainly responsible for AD, through antioxidant enzyme regulation, significantly increasing cell viability in a dose-dependent manner. Since PUFAs content, and especially DHA, is of great importance for fetal brain development [180], Balakrishnan et al. [181] examined the effect of *Isochrysis* biomass administration for 30 days prior to pregnancy (containing 5.7 mg of EPA + 1.4 mg of DHA/kg bw) to female Wistar rats, regarding their first-generation offsprings’ development. According to the results, the DHA content was significantly increased in the fetal brains of the test group (6.5 μg/mg tissue) when compared to those of the control group (3.9 μg/mg tissue), a concentration higher than that of their parents. When the offspring were treated with monosodium glutamate to stimulate neurotoxicity, *Isochrysis* supplementation exerted a neuroprotective effect, limiting histological lesions and apoptotic activity.

Microalgal PUFAs are also generally accepted as antioxidant and anti-inflammatory agents. Conde et al. [182] tested several strains’ extracts for their antioxidant activity, with an emphasis on fatty acids correlation. As shown in Table 7, except for *Spirulina*, the higher the PUFAs content the greater the scavenging activity of the extracts, with the best performing being *S. obliquus*, and the worst performing being *N. oceanica* and *T. chui*. *Schizochytrium* sp., by far the lipid-richest microalga, was studied by Zeng et al. [183], who revealed that the lipid extract of the microalga containing 28.5% wt DHA showed a strong potential for antioxidation, increased proliferation of derma papilla cells, and regulated iron homeostasis, promoting hair growth. When tested in vivo, the extract had effects similar to 5% minoxidil, a conventional drug used for limiting hair loss. This finding suggests the microalga’s ROS-scavenging extract’s possible use in treating hair loss and alopecia.

The anti-inflammatory properties of microalgal PUFAs have been researched both in vitro and in vivo. Robertson et al. [184] utilized a *P. lutheri* lipid extract rich in PUFAs (relative abundance of 51.85 ± 0.84%), especially in EPA and DHA, accounting for 27.67 ± 0.37% and 10.47 ± 0.38% of its total fatty acids, respectively. The extract’s anti-inflammatory potential was evaluated on LPS-stimulated human THP-1 macrophage cells, where it was proven to limit pro-inflammatory gene expression and inhibit the respective signaling pathways, leading to minimized cytokine production. The writers also highlighted the possible synergistic effect of the PUFAs with the existing pigments of the extract, such as chlorophyll α and β carotene, which could have enhanced the extract’s properties. Gutiérrez-Pliego et al. [185] supplemented two different strains of mice, db/db (strain with chronic inflammation) and CD1, with EPA and DHA extracted from microalgae from the Chlorophyceae and Eustigmatophyceae families, in doses of 2% of their everyday diet, resulting in a significant downregulation of inflammatory cytokine production, for both species in a similar way, even though their characteristics are completely different. The above results were also validated in a human randomized control trial conducted by Dawczynski et al. [186]. A total of 38 patients with diagnosed rheumatoid arthritis, an autoimmune disease of joint inflammation, consumed 2.8 g DHA derived from *Schizochytrium* sp./day in the form of oil-infused products for 10 weeks, which prove to be a therapeutic dose as it ameliorated symptoms of joint tenderness and edema, increased DHA concentration in the erythrocytes, and minimized disease activity markers, reinstating a balance towards the anti-inflammatory state. 

Microalgal oils have been proposed as possessing anticancer and antiproliferative effects against different types of malignancies. The treatment of breast and lung cancer cells with a *Chlorella* sp. S14 extract, rich in PUFAs (52.87% of total FAs, with 12.37% of them being ω-3 FAs) at a dose of 150 μg/mL, significantly reduced their viability in a dose- and time-dependent manner to 31.58% and 62.56%, respectively, after 48 h, while displaying no cytotoxic effects against normal neuronal cells. Especially for breast cancer cells, the extract stimulated enhanced catalase activity and minimized nitric oxide production, markers of DNA damage due to oxidative stress that has been blamed for cancer occurrence [187]. Castejón et al. [188] combined ultrasound-assisted extraction with enzymes in order to produce a *N. gaditana* extract rich in ω-3 PUFAs (30.2 ± 2.4% of EPA) which displayed a strong antiproliferative and, yet, selective effect against human colon cancer cells. 

Lipid metabolism is a metabolic pathway responsible for balancing the lipid content in blood and tissue, which has been implicated as a key factor in cardiovascular disease development, obesity, and diabetes [189]. Microalgal PUFAs and lipid extracts have been proven to possess great potential for regulating hyperlipidemia. Specifically, a meta-analysis of clinical trials carried out by Bernstein et al. [190] revealed that a median dose of 1.68 g algal DHA/day may reduce serum triglycerides in healthy individuals. *Diacronema vlkianum* was also assessed as a biomass with functional properties, by evaluating a biomass administration containing 101 mg/kg bw to mice for 66 days, resulting in higher serum and tissue content of ΕPA (ω-3) and DPA (ω-6) content, reduced serum triglycerides (TGs) and “bad” low-density lipoprotein (LDL) cholesterol, enhanced “good” high-density lipoprotein (HDL) cholesterol accumulation, and an overall improved ω-3 index and lipid profile [191]. Similar results were obtained by a three-month control trial supervised by Rao et al. [192], during which participants were supplemented daily with a capsule containing 250 mg EPA extracted from *Nannochloropsis*. The data recorded showed an ameliorated ω-3 index, a significant decline in VLDL cholesterol and total cholesterol (TC) in general, an effect more pronounced for the subgroup of participants with higher baseline values of the cardiovascular health biomarkers. Complementarily, a reduction in body weight and hip circumference was observed after 12 weeks of supplementation. Another microalga equally rich in PUFAs, *P. tricornutum*, was used as a supplement in male Wistar rats in doses corresponding to 33 mg EPA/kg/day for eight weeks, leading to ω-3 enrichment of the plasma, red blood cells, and liver tissue. The microalga biomass administration prevented hyperinsulinemia, hypertriglyceridemia, and hypercholesterolemia, restoring these respective values to those of the control group. It also ameliorated the inflammatory status of the rats and resulted in an improved body weight by inducing fat cell apoptosis.

Li et al. [193] used a 95% ethanol extract of S. platensis, rich in PUFAs, for hyperlipidemia treatment and gut microbiota regulation, and the rats were observed after supplementation with 150 mg of extract/kg/day for 8 weeks. Specifically, the TGs and TC were lowered by 49.41% and 35.68%, respectively, compared to the high-fat-fed rats, while the HDL cholesterol was increased by 50% at the end of the trial. A histopathological examination of the liver revealed the major protection of the tissue from lipid accumulation, where the *S. platensis* extract ameliorated damage and inflammation, which was confirmed by hepatic enzyme regulation. Since gut microbiota plays a key role in lipid metabolism, the extract’s effect on the intestinal flora was evaluated. Notably, the supplementation increased the beneficial bacteria, such as *Prevotella*, while it limited the presence of harmful bacteria associated with dyslipidemia, *Turicibacter*, and *Clostridium XVIII*. By following a similar scientific approach, Wan et al. [194] observed similar results after administering 55% ethanol extracts of *C. pyrenoidosa* and *S. platensis* at a dose of 150 mg/kg/day to male rats fed a high-fat and high-sucrose diet for 8 weeks. At the end of the treatment period, both microalgal extracts exhibited a hypoglycemic effect, with *C. pyrenoidosa* ameliorating to a greater degree the glucose tolerance of the rats compared to *S. platensis*. In addition, supplementation with *C. pyrenoidosa* and *S. platensis* diminished the abundance and distribution of *Blautia* and *Turicibacter* while maintaining the amount of *Oscillibacter*, *Parasutterella*, and *Ruminococcus*, among other beneficial bacteria, in the intestines. Specifically, based on the differences in gut microbiota between the *C. pyrenoidosa*, *S. platensis*, and control groups, *Ruminococcus* may very well be the principal bacteria that is the key regulator of diabetes. 

## 3. Extraction of Bioactive Compounds from Microalgae

Microalgae have great potential as a source of natural substances that are beneficial for health and can be used as food supplements. Thus, they have attracted much attention from the food industries. This, of course, requires their immediate consumption, which is why the selection of the most appropriate extraction technology for the recovery of their valuable bioactive compounds is very important. Also, the application of environmentally friendly methods with increased productivity is very important [195,196,197]. In this context, new extraction techniques, such as ultrasound-assisted extraction, are gaining more and more attention [195,198].

Extraction is one of the oldest “chemical” activities. The extraction of essential oils, pigments, or bioactive substances from plant raw materials are essentially primitive extraction processes, where the desired component is usually transferred from the plant raw material to an aqueous phase using hot water. Extraction is considered as the process by which a substance is transferred from a phase where it is either in the form of a solution or dispersion to a liquid phase. With this technique, the isolation of a substance from a mixture is obtained by its close contact with a solvent that selectively dissolves it. The starting mixture may be a solid or liquid natural material or a crude reaction mixture. Depending on the case, a different technology is applied [199,200].

As mentioned earlier, extraction is defined as the removal of a substance A from a mixture of substances using a solvent (mostly organic solvents) and is one of the most important separation methods for a wide variety of components and samples. The advantages and disadvantages of different extraction methods are presented in Table 8.

This review paper is going to focus on the most innovative, newly developed extraction techniques with the most promising and interesting attributes concerning their environmental output, cost demands and scale-up capabilities. Therefore, ultrasound-assisted extraction, microwave-assisted extraction, and pressurized liquid extraction methods are going to be further examined. 

Compared with traditional extraction methods, ultrasound-assisted extraction is a good technique. This is due to its high efficiency and effectiveness, small energy requirements, low solvent consumption, reduced extraction time, enhancement of the quality of the produced products, and reduction in the possibility of physical and chemical hazards. This method has been systematically used for the extraction of low molecular weight substances and bioactive compounds from plants and animals. The improvement of the extraction process using an ultrasound is related to the destruction of cell walls, the reduction in particle size, and the enhancement of mass transfer from the cell walls [195,198,208]. 

### 3.1. Ultrasound-Assisted Extraction

An ultrasound is defined as a mechanical oscillation/vibration of matter with a frequency above the upper audible limit (audible limit 20 kHz). The wave propagates through a solid as a disturbance of its particles, which sustains the propagation of the wave. The oscillation takes place along the direction of the wave’s propagation and creates a longitudinal wave, which is essentially not accompanied by a total displacement (net displacement) of the particles and mass transfer [202,209]. 

The mechanism of extraction includes two kinds of natural phenomena. The microalgal biomass is soaked in a solvent, with the aim of diffusing it inside the cell walls, and washing out the cell components when the walls are breached. The transfer of the extractable components to the solvent takes place through the effects of diffusion and osmosis. In particular, an ultrasound appears to facilitate swelling and hydration, thereby causing cell wall pores to enlarge. An increase in the swelling of the plant tissue can, in some cases, rupture the cell walls, favoring the leaching process. In this way, the diffusion process is facilitated and, therefore, the mass transfer is enhanced. Both of these phenomena appear to be sensitive to sonication. Ultrasounds increase the swelling index, i.e., the uptake of water into the microalgal biomass during processing. The extraction index is much higher for this process compared to mechanical agitation. The increasing swelling of the algae’s cell wall tissues leads to their rupture, facilitating the leaching process of the extractable components [210,211,212]. When a liquid is sonicated, microbubbles are created that grow and oscillate rapidly, causing them to rupture violently when the sound pressure is high enough. By definition, ultrasounds are high-frequency waves that transfer pressure during their passage through a medium. This results in the creation of areas of low and high pressure. This pressure variation is referred to as the pressure amplitude and is proportional to the amount of energy applied to a system. When the pressure fluctuations are high enough (3000 MPa), then a liquid medium can be destructured and microbubbles of gas and vapor are formed and compressed. The increase in pressure and temperature from condensation causes the bubbles to break up. This phenomenon is known as cavitation, and the bubbles can break up and be re-created continuously, causing changes in the structure of the medium that is affected by the ultrasonic waves. 

The violent bursting of microbubbles near the surface of a solid leads to the generation of microjets and shock waves. Furthermore, in the liquid phase surrounding the solid, high micromixing will increase the heat and mass transfer as well as the diffusion of the various substances within the pores of the solid [202,209,213]. Two general types of ultrasonic extraction devices are ultrasonic baths and closed-type extractors equipped with an ultrasonic transducer. The mechanical properties of ultrasounds cause the greater penetration of the solvent into the cells and improve the mass transfer. Ultrasounds can also disrupt biological cell walls, facilitating the release of cellular material. Therefore, the satisfactory disruption of the cell walls and a sufficient mass transfer are considered as the two key factors leading to the enhancement of extraction with the help of ultrasounds [214,215]. 

According to the literature, unlike other conventional extraction methods, plant extracts, including microalgae, diffuse across the cell walls in a shorter time using ultrasounds [210,211,216,217,218]. 

This extraction technique is a cheap, simple and effective alternative to other conventional extraction methods. The main advantages of using ultrasounds in solid–liquid extractions are increased extraction efficiency and faster kinetics. Ultrasounds allow for greater penetration of the solvent into the sample and increase the contact surface between the liquid and solid phase. These, combined with an increased mass transfer and significant cell disruption, through cavitation, which will be discussed below, increase the release of the intracellular product into the bulk medium [196]. Ultrasounds can also lower the operating temperature, allowing for the extraction of thermosensitive substances [219,220]. Even though heat is generated by ultrasound wave implementation, the extraction temperature is efficiently controlled by using the proper refrigerant system in combination with a sensitive thermocouple. In addition to that, the short extraction time limits the possibility of thermal degradation [221]. Compared to other innovative techniques, such as microwaves, an ultrasound device is cheaper and simpler to use. Furthermore, ultrasonic extraction, like Soxhlet extraction, can be used with any solvent to extract a wide range of natural components [210,212].

However, the effects of an ultrasound on extraction efficiency and kinetics may also be related to the nature of the raw material. In addition, the presence of a dispersed phase may contribute to the damping of the ultrasound wave, and the active part of the ultrasound within the extractor is limited to an area adjacent to the ultrasound source. Often, the violent collapse that occurs due to this transient repeated cavitation can bring about a large number of chemical changes either inside the vapor bubbles or at the interface with the liquid medium. With the collapse of such a bubble, free radicals are created and in the presence of water, primary free radicals H and OH are formed, which recombining can lead to the secondary products of the free radicals, mainly hydrogen peroxide (H_2_O_2_). Free radicals can act chemically by breaking the disulfide bonds of proteins, affecting the degree of hydroxylation of macromolecules, increasing their antioxidant capacity. Therefore, all the above factors must be evaluated very carefully when designing ultrasonic extraction devices [210,212].

### 3.2. Microwave-Assisted Extraction

Microwave irradiation is an alternative way to increase the efficiency of conventional extraction methods. Microwave-assisted extraction is enhanced by the heating of the solvent that is in contact with the sample due to the microwave’s energy. This process causes the disruption of hydrogen bonds, due to the disruption of oriented dipole molecules and the migration of ions, which enhance the penetration of the solvent into the solid, allowing for the dissolution of the extractable components [222]. 

The most important factor affecting extraction in the presence of microwaves is the dielectric constant (ε′) of the solvent. The dielectric constant is a quantity that describes the polarity of a molecule in an electric field. Usually, the higher the dielectric constant of a solvent, the higher the degree of absorption of microwaves. The solvent molecules absorb the microwave energy and become polarized. Water exhibits the highest dielectric constant among the most common solvents [223]. 

Aqueous extraction in the presence of microwaves is a “green” process in which the use of organic solvents and other chemical agents is avoided. Microwave-assisted extraction combines gentle microwave heating at atmospheric pressure, making it a particularly attractive method for isolating various components from natural raw materials. Some of the advantages of this method are the high yield of extracted compounds obtained, lower energy consumption, and the high purity of the extract [224]. Microwaves are part of the electromagnetic spectrum and lie between the range of infrared radiation and radio waves, with frequencies from 0.3 to 300 GHz. Microwaves are emitted as waves, which can penetrate materials and interact with polar molecules, such as water, to generate heat. Therefore, microwaves can heat a material uniformly throughout its mass even at the innermost points [225]. The frequency at which they are used is usually 2450 MHz, which is equivalent to an energy output of 600–700 W. At this frequency, the electric field changes the orientation of the water molecules 2.45 × 109 times per second; the chaos created inside the system prevents the synchronization with the field oscillation. This creates intense heat that can rapidly escalate, raising the temperature by several degrees per second (estimated as 100 °C/s at 4.9 GHz) [226].

Microwave-assisted extraction offers a very rapid transfer of energy to a total volume of solvent and solid plant substrate, with the efficient and homogenous consequent heating of the solvent and substrate. The water within the microalgal biomass absorbs microwave energy, leading to overheating that favors the rupture of the cell walls, facilitating the desorption of extractable components from the substrate, and improving the recovery of chromophore groups [227].

### 3.3. Pressurized Liquid Extraction

Pressurized liquid extraction (PLE), also referred to as an accelerated solvent extraction, combines high temperature and pressure to cause an exhaustive extraction of bioactive agents from a solid matrix. Due to this combined use of temperature and pressure, the mass transfer rates are enhanced while, at the same time, the solvent surface tension and viscosity are decreased and the solubility of the analytes is increased. This allows the solvent to penetrate more easily and deeper into the solid matrix being extracted. As a consequence, significantly higher extraction yields are obtained compared to conventional extractions. Therefore, PLE results not only in faster extraction processes but also in lower solvent consumption for a sample’s preparation. In addition, most of the instruments used for PLE are automated, allowing for the development of less labor intensive methods and improving reproducibility [228]. 

PLE is one of the most promising techniques for bioactive compound extraction. In PLE, the solvents are used near their supercritical region, where high temperatures produce the high solubility and high diffusion rates of the solutes in the solvent, while the high pressure, by keeping the solvent below its boiling point, enables the high penetration of the solvent in the sample. This technique is a solid–liquid extraction process performed at high temperatures (25–200 °C) with high pressures (500–3000 psi) for short periods of time (5–10 min) in solid and semisolid matrices [229]. Thus, the solvent is maintained in the liquid state during the whole extraction process. Therefore, the high temperatures increase the extraction rate through the improvement of solvation power, promoting the mass transfer of organic compounds to the solvent, and increasing the extraction efficiency. This method requires small volumes of organic solvents, in the presence of large quantities of water in the sample, providing high extraction efficiency. The parameters influencing the extraction, the pressure used and the extraction temperature, along with the extraction time, should be given consideration [228]. 

## 4. Encapsulation of Bioactive Compounds

Microalgae bioactive molecules can benefit from encapsulation in order to achieve higher bioavailability and bioaccessibility. Environmental variables can degrade bioactives generated from microalgae. Therefore, encapsulation is necessary to avoid phenomena such as oxidation and thermal degradation. Their stability and effectiveness are maintained via encapsulation, which provides a barrier against deterioration. In addition, it increases bioavailability, which guarantees the best potential absorption and function in the body. Papadaki et al. [230] manufactured ulvan—a macroalgae polysaccharide—nanofibrils containing multifunctional extracts rich in carotenoids and polyunsaturated fatty acids with ultrasound-assisted extraction, using *Haematococcus pluvialis* and *Phaeodactylum tricornutum* and coconut oil as the solvent. The encapsulation was highly efficient, reaching 90% for both bioactive components while, by adjusting the release kinetics, solubility, and interfacial properties of the lipophilic bioactives, these structurally engineered nano-systems created an efficient barrier against degrading environmental factors like water vapor, oxygen, light, enzymes, and pH. Nootem et al. [231] studied the release of astaxanthin extracted from *H. pluvialis* after being electrospun in cellulose acetate nanofibers, and compared the results to the bioavailability of the crude acetone extract. The outcomes made it abundantly evident that electrospun nanofibers are an effective drug delivery method since they can preserve astaxanthin stability while shielding it from stability testing both under controlled circumstances and in vivo. Schmatz et al. [232] managed to cover phycocyanin in ultrafine polyvinyl alcohol coatings by electrospraying, achieving a high-efficiency encapsulation (75.1 ± 0.2%), which preserved the antioxidant activities of the compound while enhancing its thermal stability up to 216 °C. Furthermore, encapsulation improves the acceptability for intake by masking unwanted tastes or odors. Singh et al. [233] developed a palatable DHA powder from microalgal oil using spray drying. The final sphere particles were manufactured with different combinations of wall materials (carbohydrates, polymers, gum, and proteins) and resulted in an encapsulation efficiency of 98.46 ± 1.1% and good oxidative stability under both accelerated and refrigerated storage settings. A two-fold increase in absorption was seen in an ex vivo intestinal permeability investigation when compared to the commercial DHA formulation. Furthermore, compared to pure DHA oil, an in vivo biodistribution investigation showed a 2.9-fold increase in brain DHA content. Therefore, this study exhibited the advantages of the encapsulation of DHA oil in order to increase its oxidative stability, shelf life, bioavailability, and organoleptic characteristics. Encapsulation is, therefore, a key strategy for maximizing the medicinal value of microalgae bioactive compounds in a variety of applications.

Polyunsaturated fatty acids are widely used in functional foods because of their positive effects on human health, in terms of coronary heart disease, and inflammatory, immune, and psychiatric disorders [21,155,234]. The unsaturation of these fatty acids makes them susceptible to oxidative deterioration and, consequently, to an undesirable taste and odor. Protection of high PUFAs lipids against oxidation is necessary to make them more stable under food processing and storage conditions [235,236]. Carotenoids are a group of natural pigments that are widely applied in food products and are characterized by a special antioxidant effect. However, due to their conjugated structure, carotenoids are highly unstable compounds that can easily degrade when exposed to oxygen or light during food storage or preparation. This can cause the loss of their nutritional and desirable properties, as well as the production of undesirable tastes and odors. For this reason, the encapsulation of these substances is necessary before their incorporation into the final product [237,238,239,240,241].

The encapsulation of substances in microstructures or nanostructures is a technology used to encapsulate bioactive components in a matrix [242]. During encapsulation, micro- or nanospheres and micro- or nanofibers are formed. The specific structures (beads or fibers) may contain hydrophobic or hydrophilic substances in a gaseous, liquid, or solid form. The particle shell (matrix) consists of a substance that serves as a “barrier” or protective mesh, which is usually a biodegradable or biocompatible polymer and determines the release rate of the active encapsulated ingredient. This barrier can either be in the form of a membrane, in which case membrane systems are formed (membrane or reservoir systems), or a polymeric or waxy matrix.

The choice of carrier is a very important parameter for the efficiency of micro/nanoencapsulation and the stability of the final product. The selection criteria are mainly based on the physicochemical properties (solubility, molecular weight, crystallinity, and emulsifying capacity), as well as the evaporation properties. Polymers made of carbohydrates, proteins, and lipid are the main classes of encapsulation carriers available [235,243,244].

Nanoencapsulation is applied with the aim of mainly improving the organoleptic characteristics (taste, smell, color, etc.) of the sensitive and/or irritating bioactive components. Also, by enclosing substances, their protection from the adverse effects of the environment in which they are found and from the processing they undergo after they are added to a product is ensured. As has been widely reported in the literature, encapsulation is mainly applied to protect components that are sensitive to heat or oxidation [245]. Also, with the appropriate selection of the medium in which the bioactive ingredient will be enclosed, its release time can be controlled to obtain the best possible result [237]. During encapsulation, the sensitive bioactive substance is enclosed in a coating medium in order to protect its biological action from external environmental factors and to strengthen its physicochemical stability. Nanotechnology has contributed to the development of systems for the encapsulation and release of bioactive substances, at the core of which a bioactive substance is dissolved into a nanoparticle size, protecting it from spoilage and degradation during the processing of the respective products, simultaneously enhancing its activity by improving its mass transfer rates [235,245,246,247,248].

The main benefits resulting from the nanoencapsulation of bioactive substances [235,249,250,251,252], and some of the main nanoencapsulation methods for bioactive substances that are used [251,253,254,255], are presented in Table 9.

In this review, based on the great advantages of nanotechnology, detailed mention will be made of the electrohydrodynamic process, which is a nanoencapsulation technology, while the innovative method of spray–freeze drying as a microencapsulation technique will also be mentioned.

### 4.1. Electrohydrodynamic Process (Electrospinning)

The method of electrospinning, or electrostatic spinning, was applied for the first time in the early 1930s. Electrospinning, therefore, is a relatively old confinement method, but interest in it has been revived in recent years, due to the development of new nanotechnologies. It is an efficient, simple, cheap, and versatile technique used to produce very thin fibers (one-dimensional nanoparticles), with average diameters between a few tens of nanometers to a few micrometers (3 to 5000 nanometers). These fibers are prepared from solutions or melts of synthetic polymers; naturally occurring biopolymers, sol-gels, and composite materials; and often contain active ingredients, such as drugs, antioxidants, and antibacterial substances; and fragrances and flavors in the form of solutions, emulsions or dispersions [256,257,258]. These nanofibers are at the center of interest due to their unique properties, such as their very large surface area per unit volume, entrapment efficiency and controlled release characteristics, light weight, tunable porosity, and design flexibility for specific physical and chemical applications. Furthermore, they are ideal for simulating the biological environment, as they have a size of the same order as that of biological molecules. In addition, they may contain active ingredients, which have many useful applications in many different fields [220].

The advantages of electrospinning, compared to other encapsulation techniques, are the following:

- It is a simpler and more flexible method with a higher containment efficiency.
- It produces very fine fibers of a few nanometers in size with a large surface area.
- There is the potential to produce nanofibers on a large scale.
- Compared to the extrusion method, the solution is not heated during the creation of the fibers, as a result of which any effects of degradation on the active ingredients from heat are limited.

The main components of an electrospinning operating system are as follows [259]:

- A stainless steel needle with a blunt tip or a capillary tube.
- A pump, whose role is to move the feeding syringe, which contains the solution to be fiberized.
- A power supply, a source of a high-voltage supply.
- A grounded collector for the fibers produced.

First, the polymer solution or melt is placed in the syringe. The basic principle of electrospinning is the application of a strong electric field. During the process, a high voltage is applied between the syringe and the substance collector and the respective solution or melt, is electrically charged. By applying a high voltage (1 to 30 kV), the polymer droplet located at the tip of the needle is highly electrified and the inductive charges are uniformly distributed over its entire surface. Subsequently, due to the electrostatic field, the droplet transforms from a spherical shape into a conical formation, the so-called “Taylor cone” [242,260]. When the voltage exceeds a specified value for each substance, the electric force “supercharges” the surface tension of the drop and, depending on the intensity of the electric field, a jet of solution is ejected from the tip of the needle. Due to the interaction of the jet, the external electric field, and the extrusion forces inside the chamber, the jet is bent and takes on a thinner shape. As the jet moves towards the target metal collector (counter electrode), the solvent evaporates and the velocity developed leads to the deposition of a layer of very fine polymeric fibers on the collector [261].

The electrospinning method and the creation of the polymer fibers depend on many factors. Specifically, they depend on the following:

- The parameters related to the properties of the solution, such as concentration, viscosity, molecular weight, surface tension, nature of the solvent, and conductivity.
- The parameters related to the device, e.g., flow rate, applied electric field strength, solution volumetric flow rate, needle–collector distance, temperature, humidity, and air flow inside the chamber, whose shape regulates and affects the fibers’ properties, such as diameter, uniformity, porosity, or even their various defects.

Therefore, the size of the nanofibers produced and, thus, their surface-to-volume ratio and porosity can be controlled by varying [262,263,264] the following:

- The composition of the solution (by changing the type of polymer, its concentration, or the type of solvent).
- The properties of the solution (by changing pH, temperature, and ionic strength).
- The device parameters (by varying the applied voltage, the distance between the source and collection surface, and the relative humidity of the chamber).

### 4.2. Spray–Freeze Drying

Spray drying is a classic encapsulation technology, which requires high temperatures, causing the thermal degradation of heat-sensitive compounds. Alternatively, freeze drying has been reported to overcome the limitations associated with this technology. However, conventional vacuum freeze driers require a long drying time due to poor heat transfer rates, leading to high energy consumption, while the particle size of the encapsulation product cannot be controlled [265]. 

In order to overcome these problems, an alternative technology, namely spray–freeze drying, was developed for microencapsulation as a combination of the aforementioned methods. During the spray–freeze-drying process, the feed solution is sprayed into droplets through an atomizer and the produced droplets are solidified by contact with a cold fluid. Finally, the frozen droplets are sublimed at a low temperature and pressure to obtain a dried powder [266,267]. 

This technique allows for a high degree of control over the residual moisture content, mass density, and particle size; in fact, it allows for the easy manipulation of the process parameters, such as the temperature of the cryogenic liquid, the chemical composition and concentration of the solution, and the choice of atomizer type [268]. In particular, the choice of the atomizer is a very critical step, which includes the selection of the appropriate nozzle and the adjustment of the nominal flow rate. Moreover, the drying conditions and the solution’s characteristics are also important parameters for the process [267].

The spray–freeze-drying technique can be categorized based on how the freezing of the sprayed feed solution is achieved. More specifically, there are the following [267]:

- Spray–freezing into a vapor, where the feed solution is sprayed into a chamber with cold gas causing the particles to freeze.
- Spray–freezing into a liquid, where the nozzle of the atomizer is placed into a cryogenic liquid and the spray occurs directly in it, producing frozen microparticles.
- Spray–freezing into a vapor over a liquid, where the atomization occurs in contact with a cold gas. The particles begin to freeze during their dispersion into the vapor and fall into the cryogenic liquid, where they become completely frozen.

This technology is mainly used for drying and encapsulating high-value components (often of low water solubility) intended for incorporation into pharmaceuticals (e.g., antibodies, vaccines) or food products [266].

## 5. Life Cycle Analysis (LCA) as a Sustainability Tool for Process Optimization 

Pioneering technologies, such as those mentioned in this review, have emerged through academic and industrial research at an exponential rate. However, within the scope of sustainability and the circular economy that climate change and environmental policy demand, it is necessary to ensure that the production process impact is limited to the absolute minimum. Identifying the environmental hotspots of a process through observation and real-time experiments is considered time-consuming and cost-ineffective, as it requires using equipment and materials without delivering any final product of use. An alternative to the scenario above is the simulation of the process through software and the comparative evaluation of the obtained data. The main tool for environmentally optimizing a process is Life Cycle Analysis (LCA). LCA is a methodology implemented to identify and quantify the impact of each stage of a the production system, from obtaining the raw materials (“cradle”) to the formulation of the final product (“gate”) and its disposal (“grave”) [269,270]. Therefore, it is considered a key factor in corporate decision making and strategic planning.

Concerns for the short- and long-term effects on the environment have risen among the scientific community as well as the public since the early 1970’s. One of the first companies to address the issue was Coca-Cola in 1969, when it ordered a study on the different resource requirements, waste flows, and emissions of refreshment containers made of several materials [269,271]. The popularity and acceptance of LCA as a tool for sustainable industrial development continued to grow in business and government policy making, mainly about packaging [271,272]. Software development, standards making, database construction, and other activities benefitted LCA, and a vibrant community of technique developers and practitioners arose in the following years. GaBi was established by PE International in 1989, and it was followed by PRé Consultants’ SimaPro (1990). As different methodologies and theoretical frameworks were used for determining the same problems, it was a usual phenomenon for researchers to come to contradicting results, a fact that highlighted the need for liability and certainty [273,274]. To facilitate collaboration among LCA practitioners, users, and scientists regarding the ongoing enhancement and standardization of the framework, terminology, and methodology, the Society of Environmental Toxicology and Chemistry (SETAC) took the lead, along with the International Organization for Standardization (ISO), which modeled the analysis for the first time in 1994 with the ISO 14040 series, and was updated continuously until 2012 [271,273,275].

### 5.1. The ISO Framework

The ISO Framework consists of four main stages when conducting an LCA report [276]:

1. Goal and scope definition.
2. Inventory analysis.
3. Life cycle impact assessment.
4. Life cycle interpretation.

**Figure 10:**
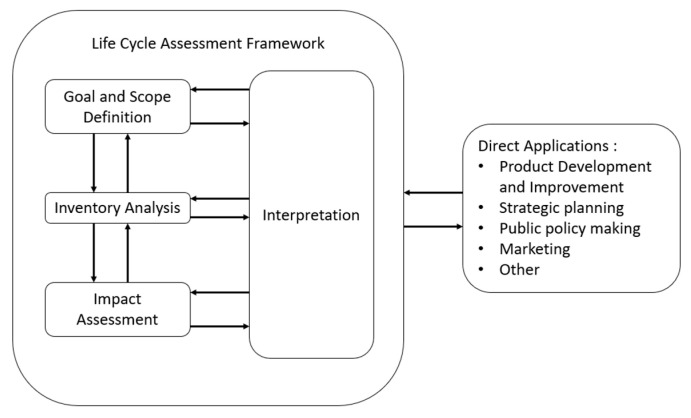
The methodological framework of LCA according to ISO [276].

The correlation between the stages and possible applications is demonstrated in Figure 10.

#### 5.1.1. Goal and Scope Definition

This section’s focus is on framing the question and outlining the context in which it should be answered. The goal should address, amongst other things, the intended application of the study, the arguments for carrying it out and, thus, the decision context, aspects that heavily influence the latter stages of an LCA report. The means of communicating the results, the intended audience, the commissioner of the study, and the availability of the results to the public for comparative studies should be mentioned. In the scope definition, the direction of the study and main parameters for conducting the LCA should be decided and well defined, ensuring and documenting the consistency of the techniques, assumptions, and data and, thus, the study’s repeatability. First of all, the process stages that contribute to the life cycle are determined, and the functional unit is established. The functional unit expresses the quantity basis which is used as the reference flow for the product for which the assessment is conducted. It is one of the key parameters, since it diminishes any differences between the process systems by normalizing their environmental impacts on the selected unit, rendering them comparable. Accordingly, the deliverables, modeling framework, assessment parameters, impact categories, and the treatment of uncertainty and methodological limitations are presented [271,273,276]. 

One of the most significant parameters are the system boundaries. There are three main types of boundaries, including the interactions between the technological system, the environment, and other technological systems, as well as the interactions between major and minor processes inside the system. The interaction with the environment is quantified both through the input and output flows of the resources and emissions. It is challenging to draw a line in the system between important and trivial activities, since it is typically unknown ahead of time which facts are considered insignificant. A processes’ whole life cycle contribution is occasionally utilized as the cut-off criterion (e.g., <1 percent of mass). However, this method might result in large underestimations since environmental effects might not be well correlated with mass. It is also necessary to establish the system’s boundaries concerning other technical systems. This is especially risky when “multifunctional processes” are part of the LCA. Multiple product systems share a multifunctional process, and it is unclear which product system should bear the environmental costs of the process [271,273,276].

#### 5.1.2. Inventory Analysis

The “phase of life cycle assessment including the collection and quantification of inputs and outputs for a product throughout its life cycle” is how the ISO defines inventory analysis (LCI). It is evident that quantification plays a significant role during this phase and that numbers—both in terms of data and computations—are crucial to the inventory analysis. The unit process serves as the foundation for the LCI. It is defined as the “smallest element examined in the life cycle inventory analysis for which input and output data are quantified”. In LCA, a unit process is viewed as a black box that transforms a set of inputs into a set of outputs. There are multiple distinct types of input and output flows, such as environmental assets, waste for processing, residuals to the environment (comprising pollutants to air, water, and soil; waste heat; and noise), and products [269,271,273,276]. 

Incorporating separate unit operations into a multifunctional system presented in a flow diagram is the key characteristic of the LCI. The model’s fundamental principle is that operations are linear. This implies that a straightforward multiplication may be used to scale the data of a unit process. Although it is a crucial step toward making the computation and data collecting feasible, the assumption of linear technology is a significant limitation on the life cycle assessment (LCA). The life cycle inventory, which is a set of quantified physical primary streams for the product system related to the supply of the service or function specified by the functional unit, is the product of the inventory analysis [269,271,273,276].

#### 5.1.3. Impact Assessment

The stage of the life cycle assessment intended to identify and assess the degree and severity of the potential environmental implications for a production chain throughout the life cycle of a product is referred to as the life cycle impact assessment (LCIA) [276].

The ISO 14040 standard demands that the first three of the five components of the impact assessment are accomplished [273,274,276]:

1. Determining impact categories that are indicative of the evaluation criteria established throughout the scope definition process. A relevant indicator and an environmental model for each impact category are used to estimate the effect of primary streams on the indicator.
2. The elementary flows from the inventory are grouped into impact categories based on how they could potentially influence the selected indicator.
3. The characterization of the impact category utilizing environmental frameworks to assess how each allocated primary flow affects the category indicator. A single measure for the impact category is used to express the characterized impact scores that are obtained. This consolidates all of the contributions into a single score that reflects the overall influence that the product system has on that category.
4. By presenting each specified score for each effect category with respect to a single reference impact for each impact category, the normalization serves to provide insight into the relative severity of each impact. The product system’s standardized impact profile, which expresses each category indicator score using the same metric, is the final product of the normalization process.
5. By grouping and possibly ranking the impact categories based on their perceived intensity, or by weighting them using weighting factors that provide a quantitative expression of each impact category’s severity concerning the other impact categories, grouping, and weighting, stimulates comparisons across the impact categories.

The impact category is the key characteristic of the impact assessment. However, the evaluation of the results heavily depends on the nature of the approach, meaning if it is centered around short-term and technical measurements or long-term casualties. Thus, two strategies are mainly followed, the midpoint and endpoint strategies, respectively. The life cycle assessment (LCA) considers the effects on public health, ecosystems, and populations’ ability to survive, including the utilization of natural resources. The LCA addresses a wide variety of ecological challenges, instead of focusing only on issues like climate change. The primary goal of using a life cycle view and considering numerous climatic challenges is to prevent responsibility shifting, which occurs when initiatives to reduce one form of environmental effect unwittingly increase another. This guarantees the assessment’s evaluation of what is commonly referred to as an “endpoint.” Because there is a complex cause-and-effect path between an emission and its influence on an organism, endpoints can be challenging to quantify. Midpoints evaluate possible effects surrounding certain environmental factors to go farther up the causation chain. The potential is estimated using midpoints. The degree of damage is estimated by endpoints. The endpoint strategy has the benefit of providing more coherent measurements, while the midpoint approach has the advantage of including fewer debatable assumptions and fewer absolute facts [271,272,273,274,275].

#### 5.1.4. Interpretation 

During the interpretation phase of the Life Cycle Analysis, both the outcomes of the inventory analysis and impact assessment are evaluated as an answer to the questions posed in the goal and scope definition, leading to conclusions about the hotspots of the process, as well as optimization recommendations concerning the decisions and assumptions made throughout the study. The interpretation sets as a main goal the robust and complete identification of major impact issues, which is complimented by further sensitivity and consistency analyses and even by comparisons to previous work published. As a result, the decision-making aspect of the LCA study shines through its sustainability scope [271,273,276].

### 5.2. Applications for Bioactive Compounds Isolation of Marine Origin

LCA is mostly used to evaluate innovative processes and compare them to existing ones from a sustainability perspective, such as the recovery of bioactive compounds of medicinal value from microalgae, a group of processes that has received growing attention from the scientific and industrial communities because of the material’s multifunctional properties. In this review, the literature was examined for LCA reports highlighting the potential of algae as a nutraceutical compounds provider and the optimization of their recovery.

In this context, the LCA study conducted by Kyriakopoulou et al. [277] underlined the superior yield of 1 kg of extract rich in β-carotene from the microalgae *Dunaliella salina* in comparison to carrots, a popular source of carotenoids in human nutrition, even though the microalgae process exhibited a higher environmental impact due to the cultivation, harvesting, and drying stages needing high electricity use. However, the significant difference between the extraction yields in combination with *D. salina*’s high carotenoid content resulted in an overall more sustainable extraction process. Moreover, by evaluating different extraction techniques, they distinguished the use of ultrasound-assisted extraction for the *D. Salina* utilization and microwave-assisted extraction for carrots as the most sustainable methods, by combining them with organic solvents suitable for food applications. Optimization research should mainly focus on minimizing the cultivation and drying costs, as a result of algae’s high moisture content and multiparametric growth conditions. Proportionately, Espada et al. [278], through a biorefinery approach, considered different extraction techniques for the optimal recovery of 1 kg of β-carotene using *D. salina*, focusing on solvent-based and supercritical CO_2_ methods. A supercritical technique was superior in terms of power consumption and greenhouse gas emissions, according to the LCA findings. Nevertheless, the technology’s low extraction yield necessitated the use of vast amounts of nutrients, whose manufacturing amplified the effects of toxic metabolites in comparison to the extraction with organic solvents. The conclusions, therefore, suggest that the inefficiencies associated with the supercritical process’s feeble extraction yield are outweighed by any possible environmental benefits. Concerning the supercritical extraction using CO_2_-expanded fluids, Wang et al. [279] demonstrated the superior sustainability of the method for extracting 1 kg of microalgae oil between different organic solvents, supercritical CO_2_ and CO_2_-expanded fluids, designing six different scenarios, half of which included common extraction methods. The solvent systems were (A) chloroform and methanol; (B) dichloromethane and methanol; (C) isopropanol and cyclohexane, with the latter half used in cutting-edge green processes; (D) supercritical CO_2_; (E) CO_2_-expanded methanol; and (F) CO_2_-expanded ethanol. Indeed, avoiding conventional organic solvents, such as chloroform, dichloromethane, and cyclohexane, minimized the environmental impact, thus rendering the supercritical CO_2_ and CO_2_-expanded fluid extractions more eco-friendly. However, the high amount of energy needed for conducting such a process provided the conclusion of a further impact occurring due to electricity generation from fossil fuels. Subsequently, novel renewable energy resources should be taken into consideration in order to optimize the environmental footprint of microalgal oil production and the overall attractiveness of the process.

As far as polyunsaturated fatty acids recovery is concerned, microalgae are considered a promising alternative to fish-derived ω-3 fatty acids. Within this scope, Davis et al. [280] examined the life cycle of producing 1 kg of ω-3 fatty acids, rich in DHA, from heterotrophic algae such as *Schizochytrium*, both in powder and liquid suspension form with vegetable oils. The LCA results showed a similar environmental impact for both products, with the major contributor to climate change being the sugar cane cultivation, to obtain the sugar needed that is the key ingredient of the nutritional medium provided for the growth of the algal biomass via fermentation. The higher sugar cane yield lowered the environmental impact in all instances, as sugar production accounts for 65% of total greenhouse gas emissions (GHG). When compared to fish oil, both algae-derived ω-3 DHA products had less of an influence on acidification and climate change. However, because of the GHG emissions from burning the sugar cane residues after the extraction of the essential nutritional compounds for microalgae growth, the impact of particulate matter is greater for goods containing ω-3 DHA from algae compared to when it is derived from fish. Therefore, process optimization should focus on sugarcane cultivation and milling. When taking into account the environmental footprint of the suspension matrix, an ethically and low-impact-sourced vegetable oil should be preferred and used. Even so, DHA production from microalgae commercialization in either powder or liquid form is still less aggravating for climate change by 30–40% when compared to the traditional fish oil production. The equally substantial ω-3 fatty acid’s, EPA’s, sustainable recovery was studied by Qin et al. [281], with the resource being the microalgae *Phaeodactylum tricornutum*. For 1 g of EPA extracted, genetic and non-genetic tools were examined for process yield optimization. Namely, these included metabolic engineering, strain adaptive evolution in a higher salinity medium, the addition of a food-waste hydrolysate rich in glucose or/and crude glycerol as an additional carbon source, as well as supplementation with the antioxidant butylated hydroxytoluene for oxidative stress limitation. While the medium’s salinity modification and the supply of antioxidants had a considerable effect on the ecological impact, the metabolic engineering and the supply of additional organic substrates were found to generate the most robust decreases in the environmental impact of the EPA production process. Overall, the environmental effect of producing EPA decreased dramatically once all the technological methods were used. The extraction solvents—particularly chloroform—and electricity accounted for most of the environmental effects of the laboratory-scale procedures, hence, they are at the forefront for future development. EPA extraction by 1 kg of dry *Phaeodactylum tricornutum*, both on laboratory and pilot scales, was also assessed through a life cycle view by Perez-Lopez et al. [282], who reached similar conclusions as Qin et al. regarding the solvents, with chloroform being the most harmful, and the electricity generation impacting the lab scale. The findings obtained at the lab scale demonstrated that the life cycle environmental performance of microalgal EPA production is significantly impacted by the amount of power required, as well as the extraction agent’s (chloroform) manufacturing process. Benefits to the environment were noted when a hexane-based substitute for chloroform was suggested. Further, three primary environmental elements were found to have an impact on the production of EPA at the pilot scale: the need for power, transportation, and the supply of the nitrogen source needed for microalgae growth. The following possibilities for improvement were put out and discussed: (a) using fertilizers based on nitrogen; (b) valuing the leftover algal paste as a soil conditioner; and (c) using anaerobic digestion to produce bioenergy from the leftover algal paste.

Microalgae have also been distinguished for their potential to produce natural pigments with high medicinal value, as metabolites in the form of carotenoids or proteins. One of the most interesting scientifically is astaxanthin, whose sustainable recovery has been widely studied by LCA practitioners. Papadaki et al. [283] comparatively assessed the effectiveness of conventional and green extraction methods for the recovery of a 1 kg equivalent of astaxanthin, in the form of oleoresin extract from the dry biomass of the species *Haematococcus pluvialis*, considering cultivation, harvesting, and extraction as the main stages of the life cycle. Since artificial lighting is considered necessary both for the growth stage and the stress-induced conditions required to transform the green *H. pluvialis* microalga into a rich-in-astaxanthin biomass, cultivation is the microalgal production stage requiring the most energy. Consequently, techniques that present with a high recovery are environmentally friendly even though they occasionally have high energy demands, since the energy utilized per unit of product is significantly less when compared to that of techniques with lower energy usage but mediocre yield. More focused research on the ideal cultivation conditions is, therefore, suggested. Concerning its high yield, affordability, limited duration, and moderate environmental consequences, ultrasound-assisted extraction has been proven to be an effective sustainable extraction technique for astaxanthin. While microwave-assisted extraction is thought to be a rapid and environmentally benign method overall, it has low yields since the long required processing times result in thermal degradation of the carotenoids. The excessive use of solvents in the maceration and Soxhlet extraction processes are very costly, time-consuming, and even dangerous, rendering them inappropriate, in addition to the Soxhlet’s effect on thermal deterioration and high energy use. The processes’ total effects on the environment have demonstrated that astaxanthin extraction primarily affects eutrophication, marine aquatic ecotoxicity, and global warming, whereas solvent recycling reduces the impact of abiotic depletion. However, when comparing astaxanthin recovery from algae with other sources used in the pigment industry, Aldaghi et al. [284] concluded that microalgae extraction is not the most sustainable process compared to bacterial bioconversion and chemical synthesis for the recovery of 1 g of astaxanthin, since its considerable energy input demand during batch fermentation is the principal cause of its elevated ecological impact. The LCA results revealed that 1 g of astaxanthin synthesized through chemical reactions has a significantly lower environmental impact for all indicators, apart from ozone layer depletion, thus making it the most sustainable one. Yet, the opportunity for astaxanthin production based on a circular economy would be facilitated by using an aquaculture side stream to develop the fish feed ingredient. Zlaugotne et al. [285] came to similar conclusions when contrasting natural algal and synthetic pigments, since the climate change impact factor has the most influence on *Haematococcus pluvialis*-extracted pigment, whereas the resource and utilization of fossil fuels have the greatest impact on the synthetic pigment’s life cycle. Except for astaxanthin, phycocyanin is a corresponding pigment–protein complex associated with chlorophyll activity during photosynthesis. Papadaki et al. [286] studied the sustainable extraction of 1 kg of phycocyanin from the cyanobacterium *Spirulina platensis*, comparing the environmental impact of different techniques and solvents on the product’s life cycle. The microalga cells were cultivated, harvested, dried, and extracted as part of the life cycle stages. When paired with the appropriate solvent and pretreatment, the ultrasound-assisted extraction’s low cost, faster delivery, and mild environmental effect could be improved to be even greener. Comparing the treatment of dry and wet biomasses, the combination of a wet biomass and organic solvents like ethanol demonstrated excellent selectivity, which resulted in a low environmental effect. At the same time, it was still greater than that of the dried biomass. Although drying requires a vast amount of energy, when combined with solvents like buffer, the process of dried biomass extraction produces extracts with a higher phycocyanin content and, surprisingly, less environmental effect.

## 6. Conclusions

In summary, the vast array of opportunities for nutraceutical production presented by microalgae stems from their rapid life cycle and diverse array of bioactive compounds. Conventional extraction techniques, despite their efficacy, frequently give rise to environmental apprehensions as a result of their substantial energy consumption and thermal hazards. On the contrary, innovative methods of extraction, including pressurized liquid extraction, microwave-assisted extraction, and ultrasound-assisted extraction, provide improved efficiency, sustainability, and selectivity. Encapsulation technologies serve to enhance the solubility of bioactives and prevent their degradation, thereby optimizing their potential for a wide range of applications.

The incorporation of Life Cycle Analysis (LCA) is of paramount importance in order to optimize processes involving microalgae, thereby guaranteeing efficiency and sustainability across the complete life cycle of bioactive products. LCA offers an all-encompassing structure for assessing the ecological consequences linked to the cultivation, extraction, utilization, and disposal of microalgae products. The integration of LCA into the decision-making processes of researchers and industry stakeholders could facilitate the shift towards a circular bioeconomy and promote environmentally sustainable practices.

Furthermore, existing research highlights the substantial ecological ramifications associated with the cultivation and extraction of microalgae. This underscores the criticality of investigating sustainable energy alternatives and refining extraction techniques. Ultrasound-assisted extraction and supercritical CO_2_ extraction are emerging as promising techniques that have smaller environmental footprints than conventional methods.

In addition, microalgae such as *Schizochytrium* sp., *Phaeodactylum tricornutum*, and *Porphyridium cruentum* present viable substitutes for several bioactive compounds, with the most notable being ω-3 fatty acids (EPA and DHA) derived from fatty fish, while also having similar environmental or even lower environmental impacts, as in the case of using strain adaptive evolution in a higher salinity medium and the addition of a food-waste hydrolysate rich in glucose or/and crude glycerol as an additional carbon source. Moreover, the investigation into the utilization of microalgae, specifically *Phaeodactylum tricornutum*, to extract natural pigments has not only yielded sources that are sustainable but also diminishes their ecological imprints, especially when the possibility of valorizing the residual algal biomass after the biorefining processing is taken into account. Microalgae can also serve as major sustainable carotenoid providers, since the strains *Dunaliella salina* and *Haematococcus pluvialis* contain significant amounts of β-carotene and astaxanthin, respectively, rendering them competitive raw materials when compared to conventional sources and methods such as carrots and chemical synthesis, with great potential for process intensification and commercialization. Last but not least, the microalgae strains richest in proteins, namely *Spirulina* and *Chlorella* sp., can fill the protein demand gap while offering medicinal benefits through phycobiliproteins, while serving the circular economy concept, since their waste can be hydrolyzed into bioactive peptides. The valorization of algal waste as a source for the accumulation of bioactive peptides could serve as a central focus of research in the coming years, since protein is abundant in these organisms.

In conclusion, technological advances in extraction and the incorporation of LCA are crucial for maximizing the environmental sustainability and potential of microalgae in the production of nutraceuticals. Sustained investigation and progress in this domain are imperative in order to exploit the advantages of microalgae while mitigating their detrimental effects on the environment, thereby making a lasting contribution towards a more sustainable future.

## Figures and Tables

**Table 1:** Carotenoid content of different microalgae strains.

Bioactive Pigment	Microalgae Strain	Carotenoid Content	Reference
Astaxanthin	*Heamatococcus pluvialis*	7% dw *	[30]
		81.38% TC **	[31]
	*Chromochloris zofingiensis*	1,5 mg/g dw	[32]
Lutein	*Spirulina platensis*	2556 μg/g dw	[33]
	*Chlorella *pyrenoidosa**	125,034.4 μg/g (trans) 27,975.3 μg/g (cis)	[34]
	*Botryococcus braunii*	74.6% TC	[31]
	*Chlorella zofingiensis*	4000 µg/g 13.81 ± 1.23 mg/g	[32] [35]
β-carotene	*Spirulina platensis*	12,510 μg/g dw	[33]
		69.5% TC	[31]
	*Spirulina maxima*	80% TC	[30]
	*Dunaliella salina*	138.25 mg/g (all-trans) 124.65 mg/g (9-cis) 10–13% dw	[30,36]
	*Chlorella zofingiensis*	0.9–3.7% dw	[30]
Zeaxanthin	*Spirulina platensis*	558 μg/g dw	[33]
	*Porphyridium cruentum*	97.4% TC	[30]
	*Chlorella *pyrenoidosa**	2170.3 µg/g	[34]
	* Chlorella zofingiensis *	7000 ± 820 µg/g	[35]

* dw: dry weight; ** TC: total carotenoids.

**Table 2:** Microalgae protein content.

Microalgae	Protein Content (% Dry Matter)	Reference
*Spirulina* (*Arthrospira*) *platensis*	46–63	[87]
*Spirulina* (*Arthrospira*) *maxima*	60–71	[87]
*Arthrospira fusiformis*	39–56	[88]
*Chlorella vulgaris*	51–58	[87]
*Chlorella pyrenoidosa*	57	[87]
*Nannochloropsis oculata*	35	[89]
*Isochrysis galbana*	27–29	[89,90]
*Phaeodactylum tricornutum*	30	[89]
*Porphyridium cruentum*	28–39	[87]
* Dunaliella salina *	57	[87]

**Table 3:** Microalgae amino acid content (g/100 g protein) [87,91,92,93,94].

Amino Acid	SP	SM	CV	CP	NO	PC	DS	WHO/FAO Requirement for Adults (mg/kg/day)
Alanine	9.5	6.8	7.9	5.08 ± 0.19	10.92 ± 0.01	6.67 ± 3.67	10.99 ± 0.32	-
Arginine	7.3	6.5	6.4	5.91 ± 0.07	5.93 ± 0.02	7.78 ± 0.29	8.16 ± 0.24	-
Aspartic acid	11.8	8.6	9.0	8.12 ± 0.16	9.14 ± 0.05	11.21 ± 0.45	9.56 ± 0.28	-
Cysteine	0.9	0.4	1.4	2.82 ± 0.06	0.19 ± 0.01	0.33 ± 0.01	1.63 ± 0.04	-
Glutamic acid	10.3	12.6	11.6	7.87 ± 0.23	10.30 ± 0.02	8.17 ± 0.29	12.41 ± 0.37	-
Glycine	5.7	4.8	5.8	9.73 ± 0.42	9.00 ± 0.01	6.86 ± 0.28	8.71 ± 0.26	-
Histidine *	2.2	1.8	2.0	1.64 ± 0.01	0.94 ± 0.01	1.11 ± 0.04	1.73 ± 0.05	10
Isoleucine *	6.7	6.0	3.8	6.20 ± 0.14	0.11 ± 0.01	5.25 ± 0.24	4.09 ± 0.12	20
Leucine *	9.8	8.0	8.8	3.44 ± 0.06	8.11 ± 0.05	5.83 ± 0.21	9.58 ± 0.28	39
Lysine *	4.8	4.6	8.4	8.14 ± 0.37	5.70 ± 0.01	5.50 ± 0.21	5.99 ± 0.17	30
Methionine *	2.5	1.4	2.2	3.30 ± 0.02	1.50 ± 0.01	2.78 ± 0.11	2.79 ± 0.08	-
Phenylalanine *	5.3	4.9	5.0	3.83 ± 0.11	5.05 ± 0.01	5.00 ± 0.20	6.98 ± 0.20	-
Proline	4.2	3.9	4.8	-	4.20 ± 0.07	2.53 ± 0.17	5.23 ± 0.15	-
Serine	5.1	4.2	4.1	2.79 ± 0.03	6.52 ± 0.01	8.11 ± 0.29	4.81 ± 0.14	-
Threonine *	6.2	4.6	4.8	3.45 ± 0.04	5.91 ± 0.03	6.25 ± 0.25	5.16 ± 0.15	15
Tryptophan *	0.3	1.4	2.1	-	1.24 ± 0.01	1.39 ± 0.05	0.18 ± 0.01	-
Tyrosine	5.3	3.9	3.4	1.22 ± 0.01	3.40 ± 0.02	4.43 ± 0.18	4.86 ± 0.14	-
Valine *	7.1	6.5	5.5	5.17 ± 0.05	3.29 ± 0.02	2.50 ± 0.10	7.23 ± 0.21	-

* Essential amino acids; SP: Spirulina platensis; SM: Spirulina maxima; CV: Chlorella vulgaris; CP: Chlorella pyrenoidosa; NO: Nannochloropsis oculata; PC: Porphyridium cruentum; DS: Dunaliella salina.

**Table 4:** Antimicrobial activity of PC/C-PC [122,123,124].

Strain	PC Concentration	Inhibition Zone (mm) (Mean ± STD)	MIC *	Formulation
*S. vitulinus*	100 μg/mL	0.33 ± 0.11	-	Cosmetic cream
	300 μg/mL	0.28 ± 0.10	-	
	500 μg/mL	0.39 ± 0.13	-	
	1000 μg/mL	0.57 ± 0.05	-	
*S. aureus*	100 μg/mL	0.48 ± 0.18	-	Cosmetic cream
	300 μg/mL	0.85 ± 0.07	-	
	500 μg/mL	0.90 ± 0.08	-	
	1000 μg/mL	1.21 ± 0.01	-	
	100 μg/ disc	9.33 ± 0.33	125 μg/mL	C-PC discs
*E. coli*	100 μg/mL	0.53 ± 0.11	-	Cosmetic cream
	300 μg/mL	0.60 ± 0.07	-	
	500 μg/mL	0.63 ± 0.04	-	
	1000 μg/mL	1.13 ± 0.32	-	
	100 μg/ disc	13.33 ± 0.67	100 μg/mL	C-PC discs
*P. acne*	10 g C-PC/100 g oleaginous base	23.4 ± 1.0	1.6 ± 0.4 mg/mL	Anti-acne ointment
	10 g C-PC/100 g water-soluble base	26.1 ± 1.2	1.5 ± 0.1 mg/mL	
*S. epidermidis*	10 g C-PC/100 g oleaginous base	21.3 ± 1.4	2.1 ± 0.6 mg/mL	Anti-acne ointment
	10 g C-PC/100 g water-soluble base	24.6 ± 1.6	1.8 ± 0.2 mg/mL	
*P. aeruginosa*	100 μg/disc	18.00 ± 0.58	50 μg/mL	C-PC discs
*K. pneumoniae*	100 μg/disc	16.00 ± 0.58	75 μg/mL	C-PC discs

* Minimum inhibition concentration.

**Table 5:** Antioxidant activity of microalgae peptides.

AA Sequence	Microalgal Source	IC_50_	Reference
Leu-Asn-Gly-Asp-Val-Trp	*Chlorella ellipsoidea*	0.2 mM (PR)	
		0.92 mM (DPPH)	[136]
		1.42 mM (HR)	
Val-Glu-Cys-Tyr-Gly-Pro-Asn-Arg-Pro-Gln-Phe	*Chlorella vulgaris* algal waste	9.8 ± 0.5 μΜ (ABTS)	
		8.3 ± 0.15 μΜ (HR)	[137]
		7.5 ± 0.12 μM (SR)	
Asn-Asp-Ala-Glu-Tyr-Gly-Ile-Cys-Gly-Phe	*Isochrysis zhanjiangensis*	Antioxidant activity against DPPH, HR, SR, AR	[138]
Phe-Ser-Glu-Ser-Ser-Ala-Pro-Glu-Gln-His-Tyr	*Arthrospira platensis*	171.47 μg/mL (DPPH)	[139]

PR: peroxyl radicals; HR: hydroxyl radicals; SR: superoxide radicals; AR: alkyl radicals.

**Table 6:** Fatty acid content of several microalgae.

Microalgae	Lipid Content (% dw)	PUFAs Content (% Total FAs)	EPA Content (% Total FAs)	DHA Content (% Total FAs)	References
*Schizochytrium* sp.	46–78	46.96	0.72	37.63	[164,165]
*Phaeodactylum tricornutum*	14.9	40.04	30.57	0.22	[166,167]
*Porphyridium cruentum*	5.3	70.05	29.47	-	[166,167]
*Isochrysis galbana*	28.68	51.62	1.03	20.44	[166,168]
*Nannochloropsis oceanica*	51.67	38.69	23.95	-	[166,169]
*Nannochloropsis gaditana*	8.1	4.69	-	-	[167]
*Nannochloropsis oculata*	15.6	36.48	31.24	0.45	[167]
*Pavlova lutheri*	31.36	29.38	17.76	7.61	[168]
*Spirulina platensis*	6.2	30.17	0.19	-	[170]
*Chlorella pyrenoidosa*	11.4	43.67	0.31	0.22	[170]
*Chlorella vulgaris*	13.3	38.30	-	0.30	[170]
*Dunaliella salina*	24.85	31.06	-	-	[171]

**Table 7:** Antioxidant activity of microalgae lipid extracts [182].

Microalgae	Lipid Content (% Dry Mass)	PUFAs Content (% of Total FAs)	ABTS Assay IC_50_ (μg/mL)	DPPH Assay IC_20_ (μg/mL)
*Tetraselmis chui*	6.5 ± 0.4	51.8 ± 6.6	40.9 ± 4.7	225.7 ± 6.9
*Chlorella vulgaris*	8.8 ± 0.7	64.6 ± 3.5	51.1 ± 3.7	50.5 ± 12.3
*Spirulina* sp.	10.7 ± 1.0	44.8 ± 1.5	38.7 ± 1.6	96.9 ± 9.7
*Scendesmus obliquus*	11.1 ± 1.1	60.1 ± 4.8	29.4 ± 1.2	89.1 ± 6.6
*Phaeodactylum tricornutum*	12.6 ± 1.2	54.3 ± 2.0	57.3 ± 4.5	75.4 ± 4.6
*Chlorococcum amblystomatis*	16.6 ± 0.9	56.7 ± 2.6	52.6 ± 4.6	58.4 ± 10.7
*Nannochloropsis oceanica*	20.9 ± 3.4	39.7 ± 3.4	101.9 ± 1.7	175.6 ± 8.7

**Table 8:** Different extraction techniques and major advantages and disadvantages [201,202,203,204,205,206,207].

Extraction Methods	Advantages	Disadvantages
Soxhlet extraction	Simple and inexpensive.	High solvent consumption; Labor and time intensive; Difficulty with automation and scaling-up.
Ultrasound-assisted extraction	Energy, solvent, and time savings; Extract quality preservation; Suitable for thermosensitive compounds.	Need for optimization to avoid overstimulation of treated materials; Filtration or centrifuge step needed.
Microwave-assisted extraction	Simple and low cost; Higher selectivity; Reduced extraction time.	Possible chemical structure modification of bioactive target compounds due to intense sudden heating; Filtration or centrifuge step needed.
Pressurized liquid extraction	Extract fractionation by pressure alterations; Solvent reduction; Lower extraction time.	High equipment cost.
Supercritical fluid extraction (CO_2_)	Exploitation of gas and liquid physicochemical properties as solvents; Time effective.	High equipment cost and capital investment; Poor selectivity towards polar compounds due to low polarity of supercritical CO_2_.
Enzyme-assisted extraction	High selectivity; Improved yield.	Expensive enzymes. Rigorous control of medium pH and temperature for optimal enzyme action.

**Table 9:** Different encapsulation techniques and major benefits from encapsulation.

Benefits from Encapsulation	Encapsulation Methods
Solubility increase	Phase separation
Storage time extension Covering unwanted odor and taste	Adsorption
Preservation of the aroma during storage	Formation of inclusion complexes
Protection against microbiological infections	Crystallization
Creation of stable and standardized components	Lyophilization/freeze drying
Protection of active ingredients from heat and light	Extrusion
Controlled release of substances	Electrohydrodynamic process (Electrospinning)
Avoiding degradation of thermosensitive compounds	Spray–freeze drying

## Data Availability

No new data were created or analyzed in this study. Data sharing is not applicable to this article.
